# Supplementary material for: Effects of management practices on the ecosystem-service multifunctionality of temperate grasslands
Source: Nat Commun. 2024 May 7;15:3829. doi: 10.1038/s41467-024-48049-y (PMC11076620; doi:10.1038/s41467-024-48049-y)
Supplement: Supplementary file 1 — Supplementary Information [file 41467_2024_48049_MOESM1_ESM.pdf]

Effects of management practices on the ecosystem service multifunctionality of temperate grasslands

Franziska J. Richter, Matthias Suter, Andreas Lüscher, Nina Buchmann, Nadja El-Benni,

Rafaela Feola-Conz, Martin Hartmann, Pierrick Jan, Valentin H. Klaus

Supplementary Information

Supplementary Figures

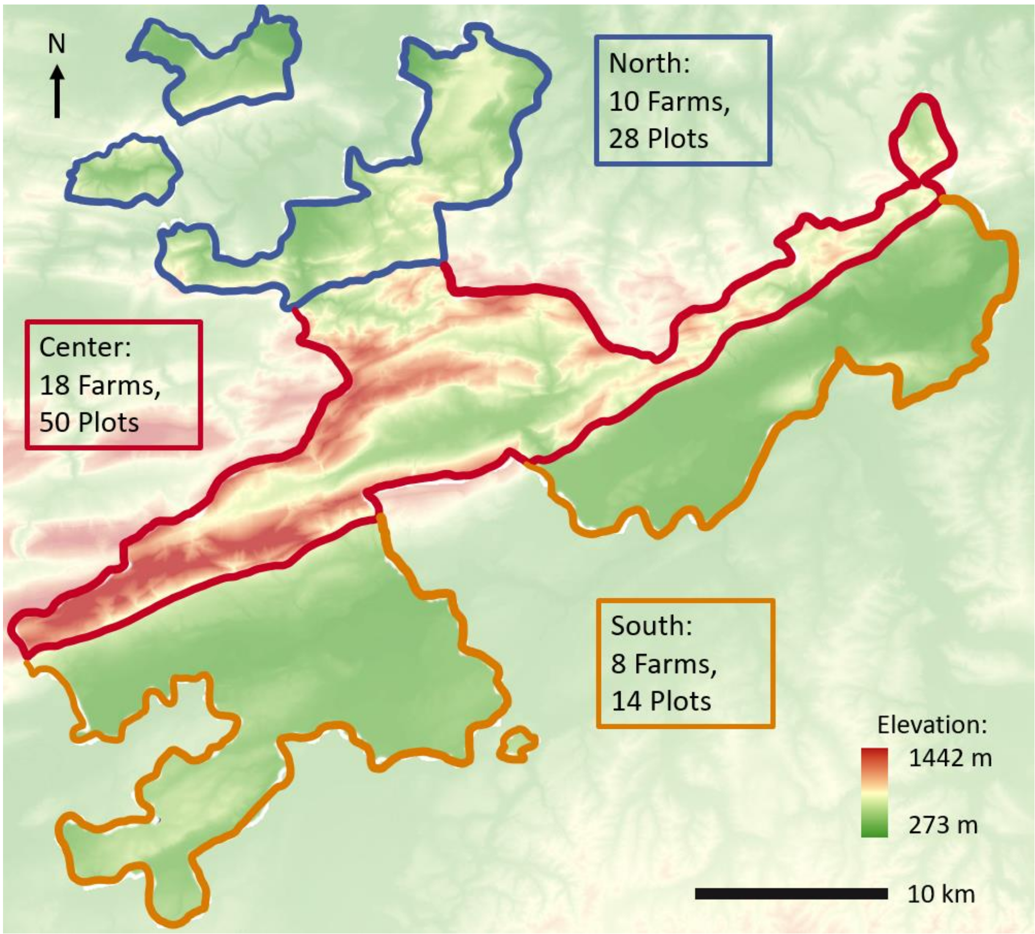

Figure S1. Elevational map of the Canton of Solothurn, indicating the number of farms and grassland plots per area within the Canton. The region is split into three biogeographical areas, which were used to representatively sample grasslands at this scale. The map does not show the exact locations of the plots to protect farmers' privacy rights. The digital elevation model used for the elevation information from European Union (2018).

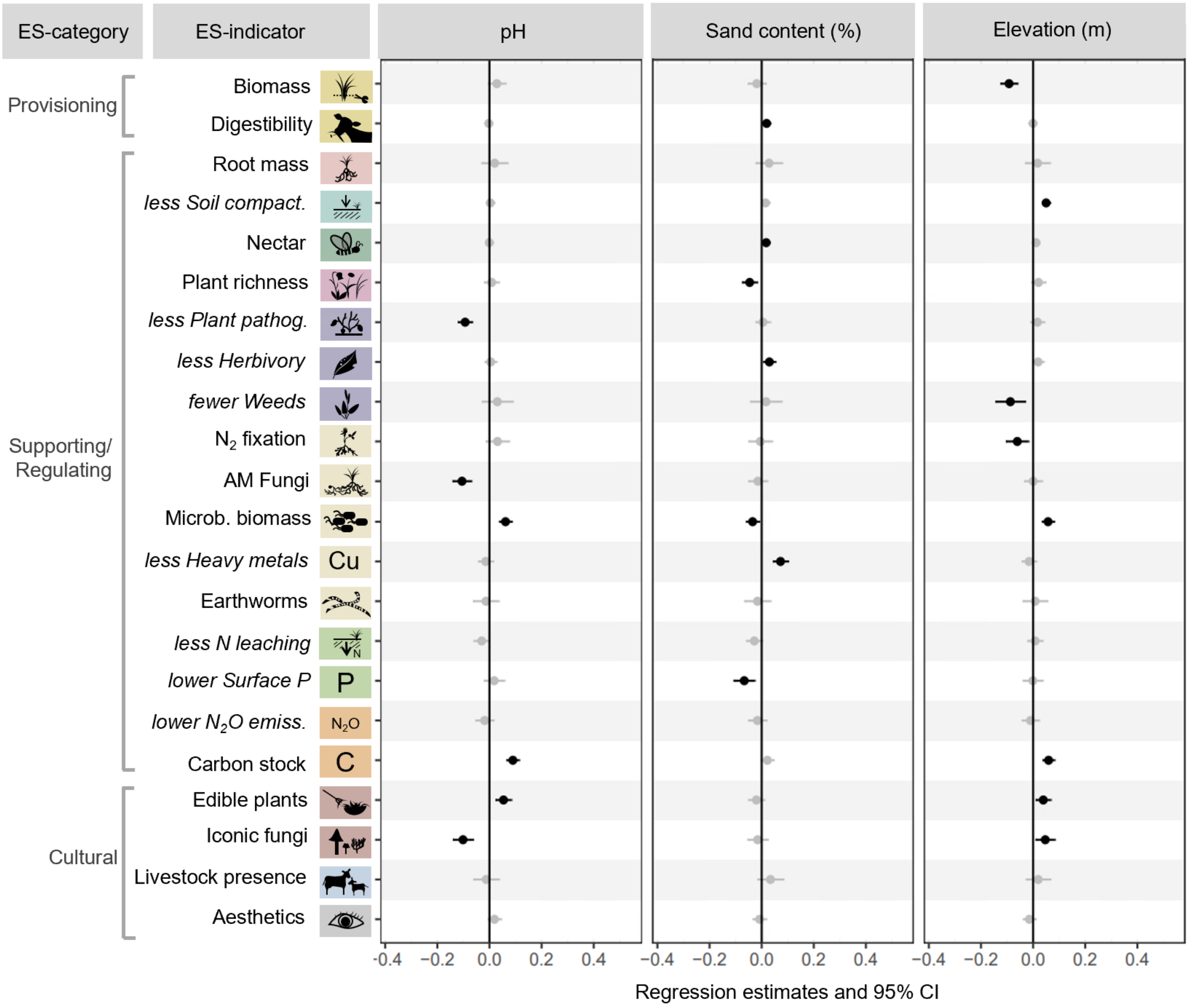

Figure S2. Effects of environmental variables pH, sand and elevation (in standard deviation scale) on 22 ecosystem service-indicators from the generalized linear latent variable model of which the effects of the management aspects are shown in the main text, **Figure 3** (Model b, **Suppl. Table S2**). Regression estimates (points) and 95% confidence intervals are shown, with significant effects shown in black ($p<0.05$). Colors of icons for ES-indicators correspond to the respective CICES-ES grouped according to the ES-categories (see **Figure 1**, main text). ES-indicators in italics have been reversed to show services instead of disservices. N = 86 grasslands.

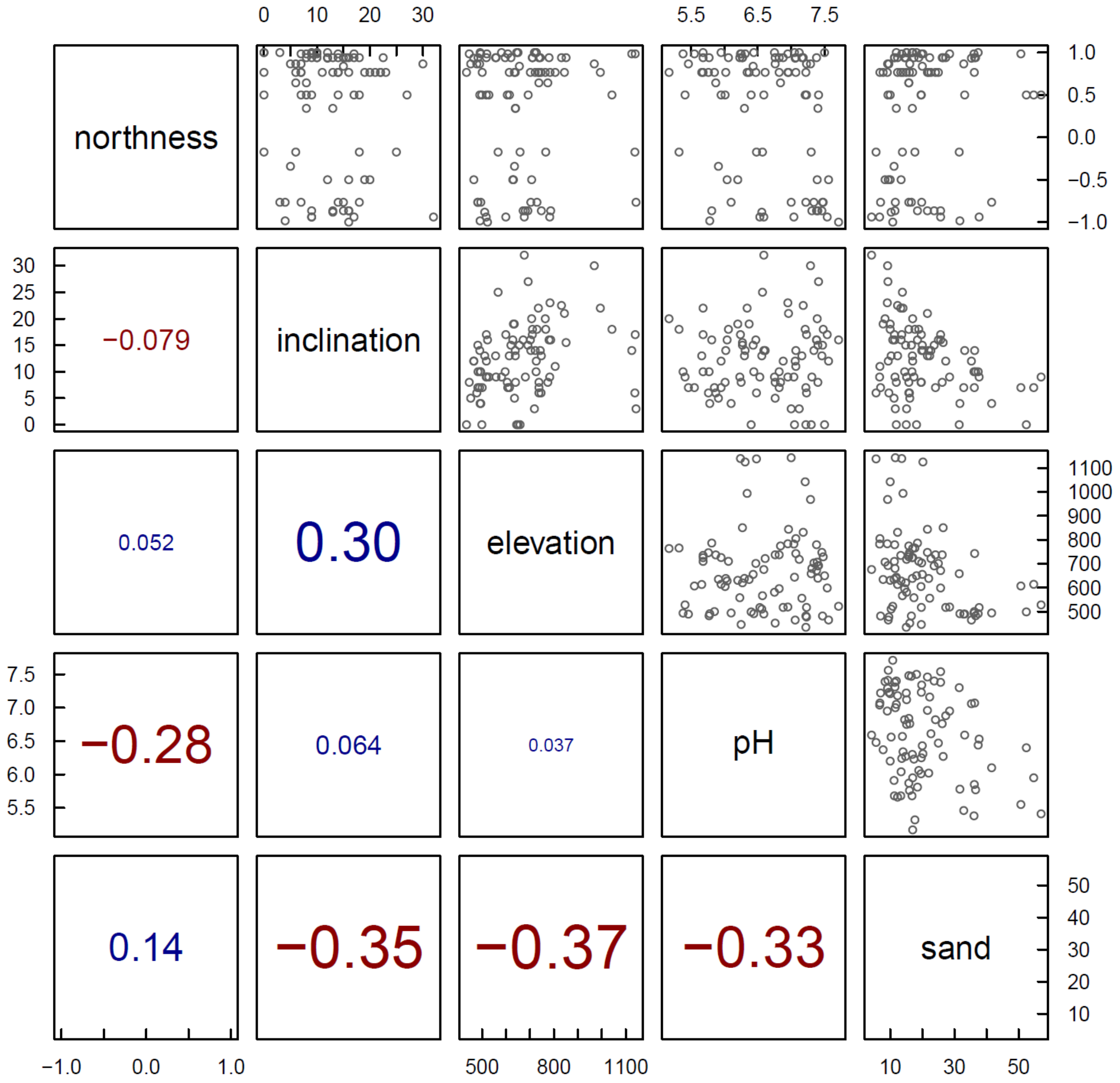

Figure S3. Pearson correlation coefficients and scatterplots showing the relationship between the environmental variables used in this study. Variables are shown on standard deviation scale. N = 86 grasslands.

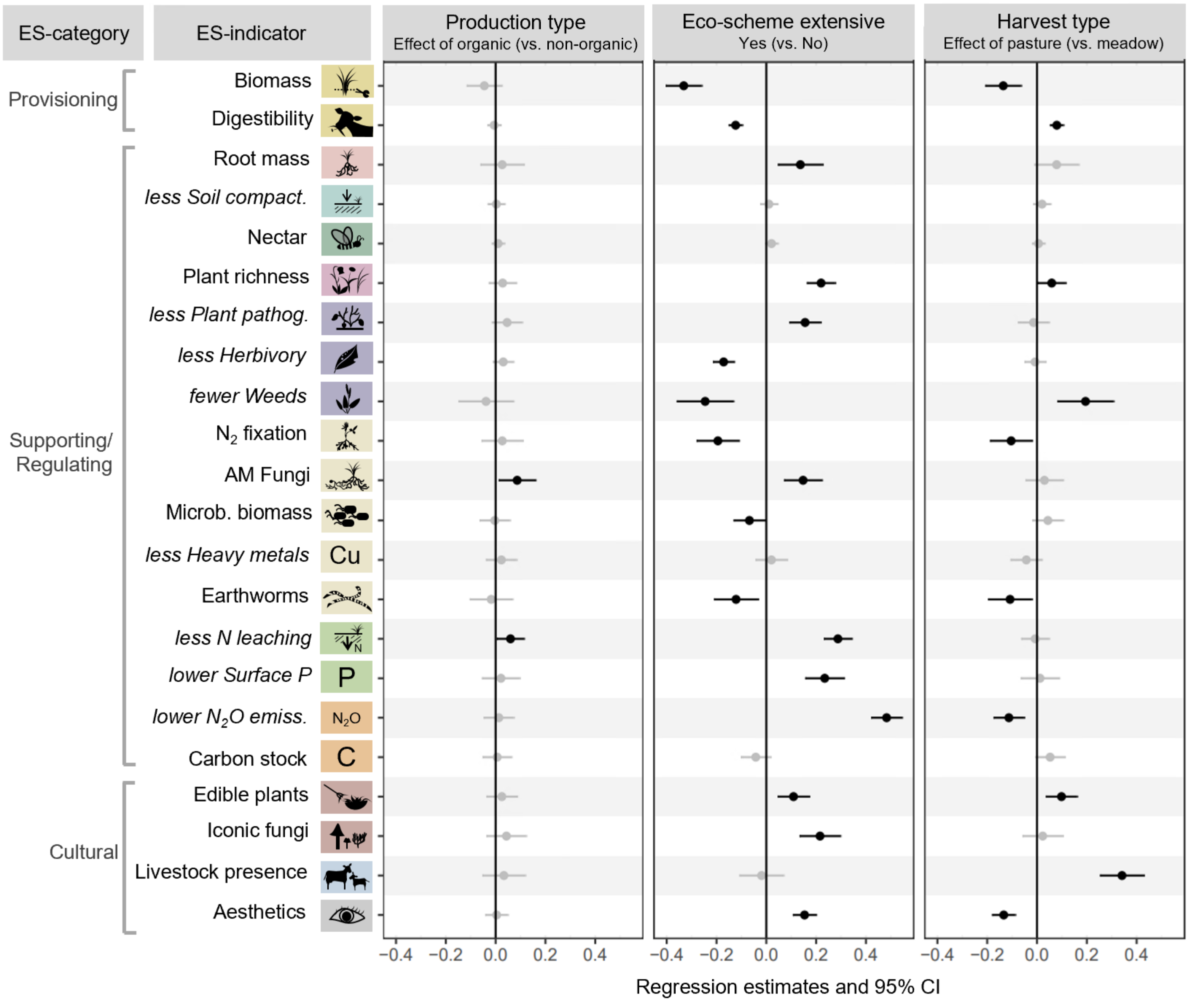

Figure S4. Effects of the management aspects Production System, Eco-scheme extensive management, and Harvest type on 22 ecosystem service-indicators from a generalized linear latent variable model (GLLVM) without environmental variables (Model a, **Suppl. Table S2**). Regression estimates (points) and 95% confidence intervals are shown, with significant effects shown in black (p<0.05). Colors of icons for ES-indicators correspond to the respective CICES-ES grouped according to the ES-categories (see **Figure 1**, main text). ES-indicators in italics have been reversed to show services instead of disservices. N = 86 grasslands.

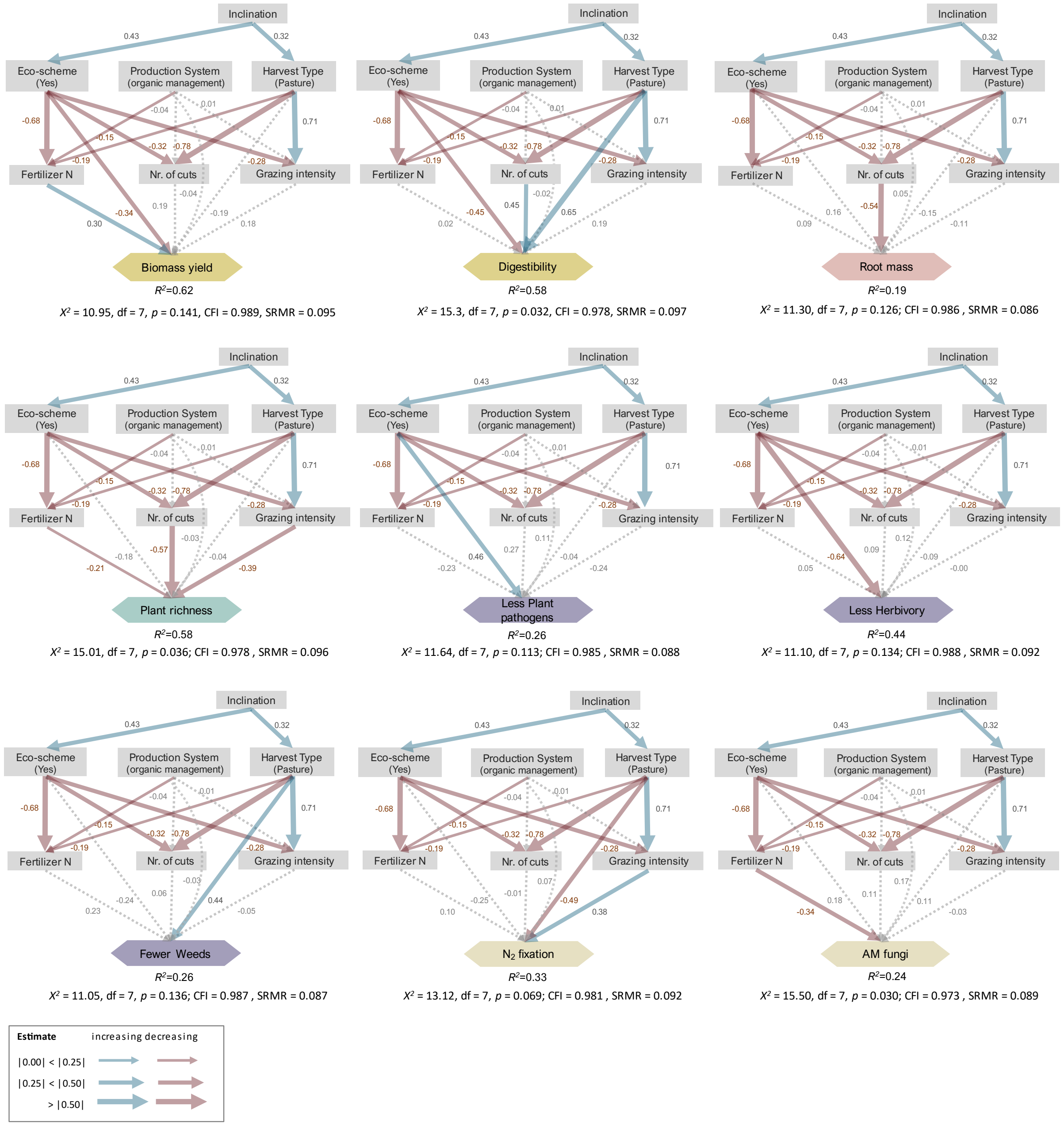

Figure S5. Structural equation models (SEMs) and corresponding fit-statistics describing the direct influence of the three management aspects Production system, Eco-scheme and Harvest type, and the indirect influence of these three aspects via fertilizer N (available N, kg ha^{-1} year^{-1}), number of cuts (cuts year^{-1}) and grazing intensity (livestock units days ha^{-1} year^{-1}). Red arrows denote negative and blue arrows positive effects, and grey dotted lines non-significant paths (p>0.05). χ^2 statistic, comparative fit index (CFI) and standardized root-mean squared residual (SRMR) of the models are given. Only the 17 ES-indicators that were significantly affected by at least one of the three management aspects as revealed by the GLLVM (**Figure 3**, main text) were included in this analysis. *Figure continued on the next page*, and see **Figure 4**, main text, for a summary of the results. Note that elevation was included in the initial model but was removed because it neither significantly affected the three management aspects nor the three measures of management intensity, i.e., fertilizer N, cutting, and grazing intensity. N = 86 grasslands.

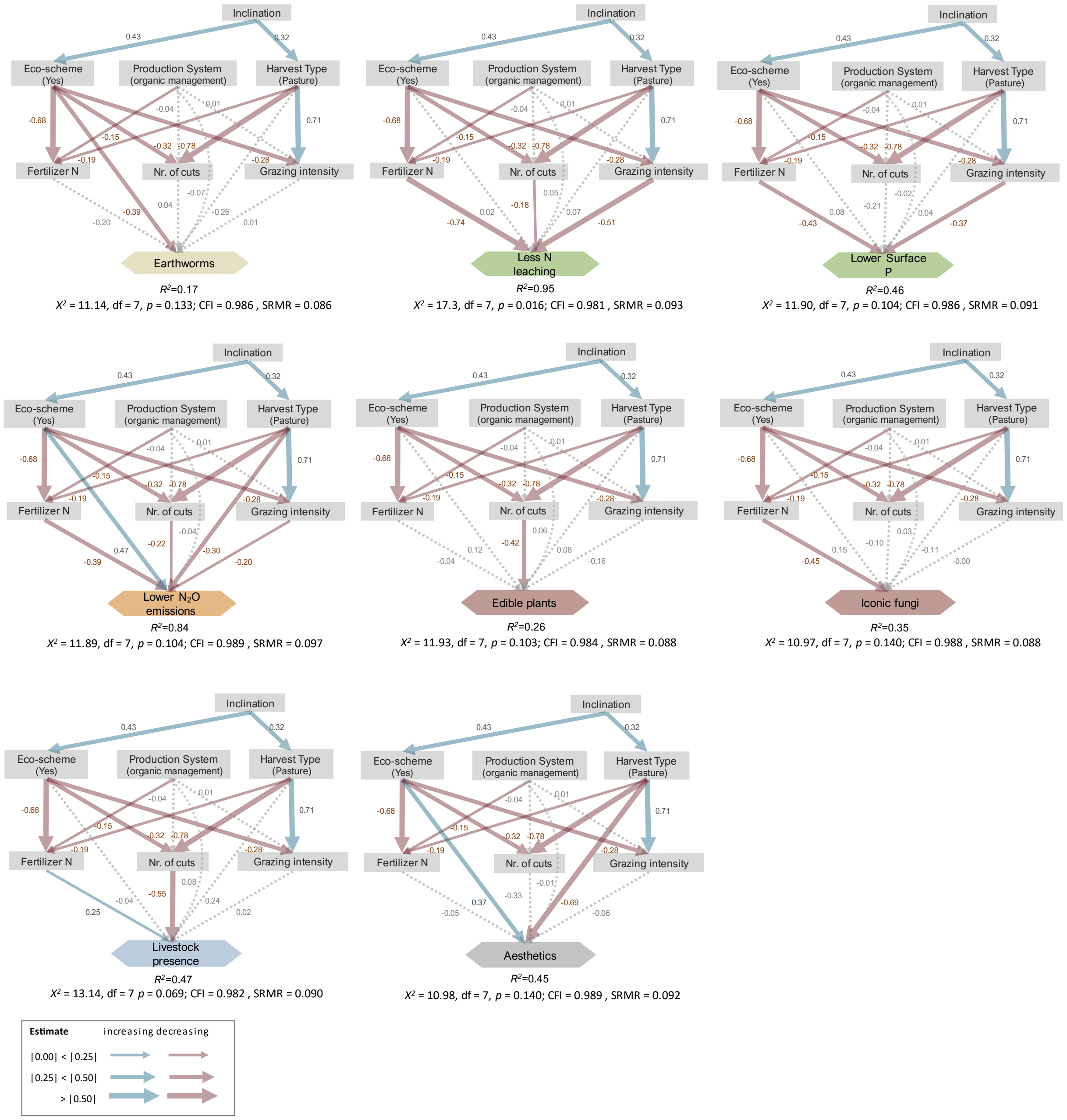

Figure S5 *continued*

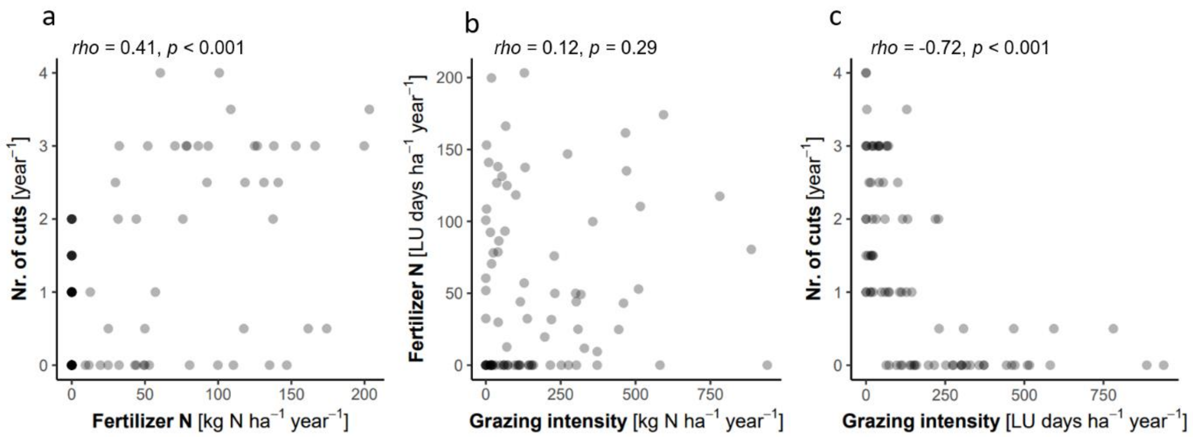

Figure S6. Relationships between management variables, i.e., a) available N in applied fertilizer (sum of organic and mineral), b) number of cuts, and c) grazing intensity (per year) along with spearman rank correlation. N = 86 grasslands.

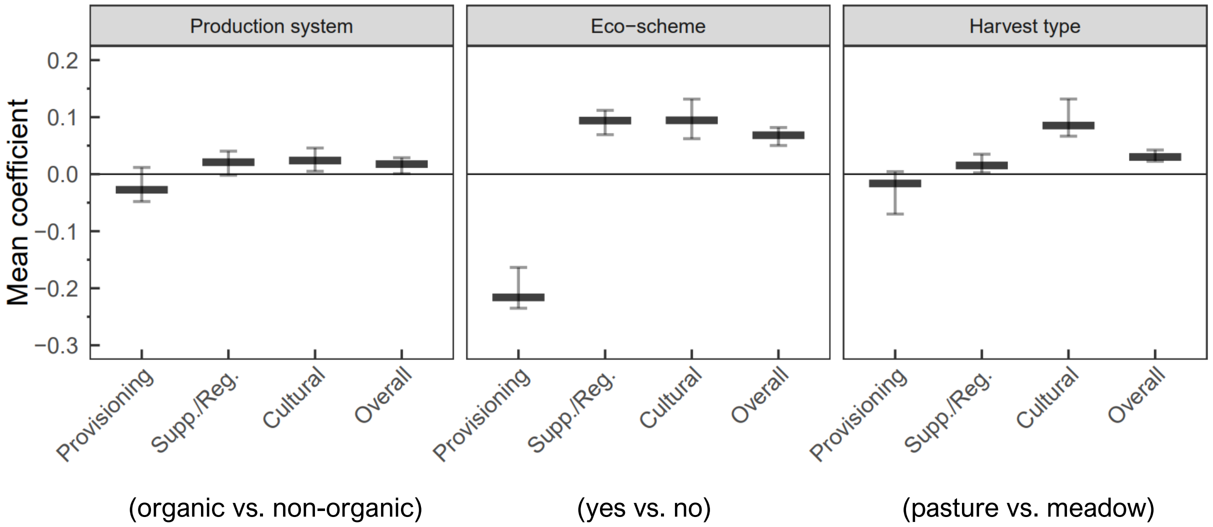

Figure S7. Effects of grassland management on multifunctionality derived by aggregating the estimates of the GLLVM displayed in Figure 3, main text. Effects were separated into overall multifunctionality (displayed in the shaded areas) and multifunctionality of each of the three ES-categories, i.e., provisioning, supporting/regulating, and cultural. Error bars represent 95% confidence intervals based on bootstrapping. The calculation of these multifunctionality indices followed the same procedure as described for the MLRR. N = 86 grasslands.

Supplementary Tables

Table S1. Mean values (and standard deviation) of management, topographical, and soil variables of the 86 sampled grassland plots grouped by Production System (organic vs. non-organic), Eco-Scheme, and Harvest Type (pasture vs. meadow).

		Organic				Non-organic			
		**Extensive**		**Intensive**		**Extensive**		**Intensive**	
		**Pasture** *N=6*	**Meadow** *N=11*	**Pasture** *N=12*	**Meadow** *N=14*	**Pasture** *N=6*	**Meadow** *N=11*	**Pasture** *N=12*	**Meadow** *N=14*
**Management**	$N_{available}$ in fertilizer – organic and inorganic N combined (kg ha^{-1}, *mean 2020, 2021)*	0.0 (0.0)	0.0 (0.0)	46.3 (52.4)	82.6 (42.8)	0.0 (0.0)	0.0 (0.0)	75.7 (55.8)	111.6 (55.7)
	$N_{mineral}$ as inorganic fertilizer, subset from above (kg ha^{-1}, *mean 2020, 2021)*	0.0 (0.0)	0.0 (0.0)	0.0 (0.0)	0.0 (0.0)	0.0 (0.0)	0.0 (0.0)	16.4 (20.9)	22.0 (21.8)
	**Grazing intensity** (LU days ha^{-1}, *mean 2020, 2021)*	181.5 (86.8)	48.3 (50.0)	409.3 (199.7)	66.0 (73.9)	171.0 (113.5)	23.3 (26.1)	435.5 (239.6)	53.5 (51.0)
	**Pesticide use** *(punctual application, number of plots)*	0	0	0	0	0	0	3	5
	**Mechanical weeding** *(number of plots)*	6	6	11	12	5	5	7	4
	**Cuts per year** *(mean 2020, 2021)*	0.0 (0.0)	1.4 (0.4)	0.0 (0.1)	2.6 (0.8)	0.0 (0.0)	1.3 (0.4)	0.3 (0.6)	2.8 (0.9)
**Topography**	**Elevation** (m)	756 (128)	615 (106)	660 (220)	657 (180)	771 (119)	627 (112)	662 (199)	682 (168)
	**Northness** (index)	0.57 (0.57)	0.11 (0.87)	0.31 (0.80)	0.35 (0.80)	0.62 (0.75)	0.09 (0.94)	0.17 (0.79)	0.42 (0.73)
	**Inclination** (°)	21.2 (4.2)	11.5 (5.1)	11.3 (4.2)	9.1 (5.1)	21.8 (7.8)	14.2 (4.7)	12.0 (5.5)	8.4 (7.1)
**Soil cond.**	**pH** *0-20 cm*	6.5 (0.6)	6.1 (0.7)	6.7 (0.6)	6.8 (0.5)	6.6 (0.8)	6.3 (0.9)	6.8 (0.5)	6.7 (0.5)
	**Sand** *0-20 cm* (%)	13.2 (3.7)	23.4 (13.6)	24.7 (14.4)	17.9 (6.4)	17.0 (11.4)	21.6 (15.5)	22.6 (13.2)	17.6 (6.5)

Table S2. Different generalized linear latent variable models calculated for this study along with the corresponding second-order Akaike Information Criterion (AICc). Table cells are shaded from white to black according to the AICc value: the lower the AICc the darker the shade. Model b, including the three environmental variables pH, sand, and elevation, had the lowest AICc value, indicating greatest parsimony, and estimated coefficients from this model are shown in **Figure 3** and **Suppl. Figure S2**. Additional models 1 - 4 at the bottom of the table, including either all five environmental variables or the two-way interactions between the management aspects are given for comparison.

Model	Formula	AICc
a	Prod_system + Eco_scheme + Harv_type	-2087.9
b	Prod_system + Eco_scheme + Harv_type + pH + sand + elevation	-2271.3
c	Prod_system + Eco_scheme + Harv_type + pH + sand + elevation + Prod_system × Eco_scheme	-2242.4
d	Prod_system + Eco_scheme + Harv_type + pH + sand + elevation + Prod_system × Harv_type	-2229.7
e	Prod_system + Eco_scheme + Harv_type + pH + sand + elevation + Eco_scheme × Harv_type	-2264.6
1	Prod_system + Eco_scheme + Harv_type + pH + sand + elevation + northness + inclination	-2218.5
2	Prod_system + Eco_scheme + Harv_type + Prod_system × Eco_scheme	-2057.8
3	Prod_system + Eco_scheme + Harv_type + Prod_system × Harv_type	-2048.1
4	Prod_system + Eco_scheme + Harv_type + Eco_scheme × Harv_type	-2096.6

Table S3. Information on the 22 ES-indicators. Overview of units in which ES-indicators were measured and the corresponding maximum value measured per indicator, as well as an indication as to whether the values were reversed in the following analysis (individual values subtracted from maximum of respective variable) for disservices.

	ES-indicators	Unit	Max	Values reversed for disservices
Provision.	Biomass	g m^{-2} °C Temp Sum^{-1}	2.44	
	Digestibility	g kg^{-1} Dry matter	772	
Supporting/regulating	Root mass	g 19.6 cm^{-2} Soil to 0.5 cm depth	6.76	
	Soil compaction	g cm^{-3}	1.52	X
	Nectar	kg ha^{-1} $year^{-1}$	222.5	
	Plant richness	Nr. species 4 m^{-2}	67	
	Plant pathogens	% Fungal ASVs, weighted average	27.5	X
	Herbivory	% Leaves damaged	0.782	X
	Weeds	Nr. individuals along 2 2×20 m transects	1002	X
	N_2 fixation	N g m^{-2} °C Temp. Sum^{-1}	0.00583	
	AM Fungi	% Fungal ASVs, weighted average	2.74	
	Microb. biomass	mg kg^{-1}	4611	
	Heavy metals	% Cu or Zn of reference value (highest value)	1.79	X
	Earthworms	Nr. individuals 0.2×0.2×0.3 m Soil Blocks	18.9	
	N leaching	kg N ha^{-1} $year^{-1}$	40.6	X
	Topsoil P	mg kg^{-1}	85.7	X
	N_2O emissions	kg ha^{-1} $year^{-1}$	3.04	X
	Carbon stock	g 100 cm^{-3}	8.15	
Cultural	Edible plants	Nr. species 4 m^{-2}	17	
	Iconic fungi	% Fungal ASVs, weighted average (CHEGD species)	37.9	
	Livestock presence	Days $plot^{-1}$ $year^{-1}$	173	
	Aesthetics	Average rating (1-5)	4.58	

Supplementary Methods

Calculating the matrix of ES-indicators for the multivariate analysis

For the multivariate analyses of the effects of management aspects on ES-indicators, the matrix of 86 plots × 22 ES-indicators was prepared applying the following four steps: To attain a multivariate normal distribution of residuals, the following indicators were natural log transformed: topsoil P content, nitrogen leaching, weed individuals, N_2O emissions, iconic fungi, livestock presence, N_2 fixation, nectar provision, and edible plants. For those indicators containing zeros, a small constant based on the variables' interquartile range of non-zero values was added prior to the log transformation, following the recommendation of Stahel (2002, p. 30) (step 1). ES-indicators containing negative values after log transformation were shifted to a positive range by adding their most negative value (step 2). This shifting does not affect the inference to management aspects drawn from the multivariate regression model. Subsequently, all ES-indicators where standardized by dividing by their maximum value (step 3). Finally, ES-indicators, for which the indicator value was inversely related to the ecosystem services provision (namely soil compaction, plant pathogens, herbivory, weeds, heavy metals, N leaching, topsoil P, N_2O emissions) were reversed by subtracting the respective indicators' maximum from its values (step 4).

Calculating the mean log response ratio (MLRR) and its confidence interval

Here, we used the matrix of 86 plots × 22 ES-indicators without log transformation and standardization (note that log response ratios (LLRs) are not affected by standardization with the maximum value). The only a priori transformation was the replacement of zero values: for those indicators containing zeros, a small constant based on the variables' interquartile range of non-zero values was added, as noted above. Calculation of LRRs was always based on the mean ES-indicator performance of the 8 management groups resulting from the 2 × 2 × 2 combinations of management aspects (organic/non-organic, Eco-scheme extensive yes/no, pasture/meadow). Thus, for example, regarding the LRR of organic versus non-organic management, an LRR-value was first calculated comparing each of the four mean performances of Eco-scheme × Harvest type combinations within organic with those of non-organic management, and these four LRR-values were then averaged to result in one LRR per ES-indicator. As noted in the main text, LRRs of ES-indicators for which the indicator value was inversely related to the ecosystem services provision (see above) were then multiplied by -1. Next, ES-indicators representing the same CICES-ES [2] (for example, *less topsoil P* and *less N leaching* for the ES 'Chemical composition of freshwaters', Figure 1a, main text), were pooled by averaging (see main text for the justification). From these values, the mean was calculated (termed mean log response ratio, MLRR) across all CICES-ES groups as well as for each of the three categories 'provisioning', 'supporting/regulating', and 'cultural' to highlight potential tradeoffs among them. An analogous procedure was applied to calculate a MLRR for Eco-scheme extensive yes versus no and pasture versus meadow.

Regarding the bootstrap procedure, the very same principle of calculation was used. Thus, we sampled 1000 times with full replacement from the replicate plots within the 8 groups of management aspects and calculated the LRRs as outlined above to receive a distribution of MLRRs for each comparison (e.g., organic versus non-organic, etc.), from which the 95% confidence intervals were calculated (following Davison and Hinkley, 1997, p. 194).

To support our results to overall multifunctionality based on the MLRR, we evaluated the effects of the management aspects (organic vs. non-organic, Eco-scheme extensive yes vs. no, and pasture vs.

meadow) on multifunctionality using two further methods. First, we calculated a mean multifunctionality index based on the estimates of the GLLVM as shown in **Figure 3**, main text. These regression estimates are the effects of the management aspects per ES-indicator and thus, an average of estimates can be interpreted as an overall effect. An average per CICES-ES was calculated from the regression estimates to down-weight indicators contributing to the same CICES-ES, followed by calculating a mean value across all CICES-ES and for each of the three CICES-ES groups 'provisioning', 'supporting/regulating', and 'cultural'. Thus, all steps in these calculations followed the same procedure as applied for the MLRR, including the calculation of 95% confidence intervals to the mean indices by bootstrapping. The result is given in **Suppl. Figure S7** (without indicating individual CICES-ES), and the outcome is almost equivalent to the MLLR (compare **Figure 5**, main text), with the exception of slightly more positive values for the effect of Production system (organic versus non-organic). Differences to the MLRR can be explained in that i) the model estimates of the GLLVM take into account effects of the environmental variables, the latter being modelled as co-variates, and ii) averages of the regression estimates are calculated from log-transformed and non-transformed ES-indicators (see above: '**Calculating the matrix of ES-indicators for the multivariate analysis**'). While i) can be seen as an advantage, ii) must be seen as a disadvantage compared to the MLRR, where all ES-indicators are treated the same by taking the LRR from the data without major transformations. Second, we calculated multifunctionality using the averaging approach and found the results to be very similar to the MLRR (no figure shown). Based on these similar outcomes from two alternative approaches, we conclude that our results on the MLRR are not only well interpretable but also highly reliable.

Replacement of missing values

The data matrix of 86 plots × 22 ES-indicators contained seven missing values (NAs) out of 1892 values, equivalent to 0.4% of the data (five NAs in the variable biomass yield, and one each in digestibility and aesthetics). Because the applied multivariate regression model (GLLVM; Niku et al. 2019) did not allow for NAs, these were replaced by a most plausible value based on the multivariate distribution of all ES-indicators and the management aspects using the R package mice [5]. For each missing value, we used the respective median of a sample of 30 possible values generated by the default method of the mice function. The imputation was done prior to the scaling steps described above (Section 'Calculating the matrix of ES-indicators').

Plot selection

We used agricultural census data from the Canton of Solothurn [6] to find possible grassland plots. In a first step, relying on insights from the definition of the typology of the Swiss Farm Accountancy Data Network [7], we created a subset of farms with a focus on grassland-based dairy systems using the criteria described hereafter. Farms had to have utilized agricultural area larger than two ha and at least 10 livestock units with at least 75% of these being cattle. The share of arable land could not exceed 70% of the farms` total utilized area. This subset of farms of the agricultural census represented the farm population that was further investigated. To ensure a non-biased representation of this population in our sample in terms of intensity and elevational gradient, we subdivided the population into four strata defined as a combination of production system (organic versus non-organic) and agricultural region (valley/hill area versus mountain area): organic in valley/hill area; non-organic in valley/hill area; organic in mountain area; non-organic in mountain area. The sampling procedure and, more precisely, the selection of nearby pairs of organic and non-organic farms, relied on these strata,

which enabled a selection of representative farm intensity levels from both valley/hill areas as well as mountain areas. The selected farmers were contacted by phone and asked for their willingness to participate. This procedure led to the final farm sample consisting of 18 pairs of organic – non-organic farms, 36 farms in total, with 16 farms located in valley/hill areas and 20 located in mountain areas. For both farms of each organic – non-organic farm pair, we selected grassland plots of the same management regarding Eco-scheme extensive and Harvest type. Yet, not all farms had each management type, resulting in a maximum of four plot-level comparisons per pair. The plots were identified using maps of agricultural census data, with each grassland having a minimum area of 0.25 ha. Plot selection to build representative pairs of organic vs. non-organic farming was based on three criteria, i.e., (i) both farms had to have the same grassland types as specified above, (ii) the grassland plots had to have a similar exposition and topography, and (iv) the grassland plots had to be as close as possible (max. within-pair distance 2 km). This finally led to 90 grassland parcels to be surveyed. After inquiring management details with farmer interviews, four plots had to be excluded as their management was not consistent with the grassland type as specified by the agricultural maps. The final dataset included 86 grasslands plots, each with one of the eight possible combinations of Production system, Eco-scheme, and Harvest type, spanning an elevational gradient from 435 to 1145 m a.s.l. See Table S1 for the exact number of replicate plots for each combination of grassland management aspects. The predominant mode of grazing for pastures was with cattle, although some plots were grazed by goats or horses, in line with the Switzerland-wide dominance of cattle compared to other grazing animals.

Measuring ES indicators: Sampling June 2020

Sampling design for heavy metals, organic carbon, microbial biomass carbon, AM-fungi, and plant pathogens, iconic fungi: samples were taken per plot. 20 soil cores were taken to a depth of 20 cm, including organic top layer, along two 18-m transects and subsequently pooled.

Heavy metals: To assess this aspect of soil health, a subsample of the air-dried soil of each plot was analyzed for copper and zinc concentrations by ICP-OES (5110 VDV ICP-OES, Agilent, Santa Clara, CA, US) according to DIN EN ISO 11885:2009-09) after aqua regia extraction (21 ml, according to DIN 38414-7:1983-01) of 5 g ground dry soil at the laboratory of the LUFA NRW (Münster, Germany).

Soil texture (i.e., clay, silt, and sand fractions) was determined by first removing the organic matter of air-dried soil samples with hydrogen peroxide using a SP 50 Robotic Analyzer. Then, soil fractions were measured with a SP 2000 Robotic Clay Fraction Analyzer (both Skalar Analytical B.V., Breda, The Netherlands).

Organic carbon stocks: Soil organic carbon (SOC) was measured via sulfo-chromic oxidation [8] of air-dried soil, and SOC stocks were calculated by multiplying the carbon concentration with bulk density (5-10 cm), measured as described below. As local carbon stocks were influenced significantly by soil clay content, we used a linear regression regressing soil carbon stock against clay content and grassland type (combination of meadow/pasture and Eco-scheme yes/no) to correct for clay content within each grassland type. Organic management and interaction terms were excluded from the model, as these terms were not significant.

Microbial biomass carbon: 100 g of fresh soil sieved to 2 mm was refrigerated, and microbial biomass **carbon** was determined via fumigation at the Federal Research and Training Centre for Forests, Natural Hazards, and Landscape, Vienna, Austria [9,10]. For this, soil samples were fumigated with ethanol-free chloroform (CHCl3) and both fumigated and unfumigated subsamples were extracted with 0.5 M potassium sulphate (K2SO4) in a 1:5 soil:extractant ratio, shaken for 30 minutes, and filtered with N-

free foldable filters (Sartorius AG, Qual. Grade 3hW, Göttingen, Germany). Dissolved organic carbon (DOC) in the extracts were measured using a TOC/TN Analyzer (TOC-L, Shimadzu, Kyoto, Japan). For microbial biomass carbon, the difference between fumigated and non-fumigated DOC was corrected by the experimentally determined extractable part of microbial biomass carbon.

Plant pathogens, AM-fungi, Iconic fungi: Fresh soil was sieved to 2 mm and frozen at -20 °C. DNA was extracted from 0.25 g soil with the DNeasy PowerSoil Pro Kit, according to the manufacturer's instructions using the QIAcube system (Qiagen, Hilden, Germany). Quality as well as quantity of DNA were measured via UV/Vis spectrophotometry with the QIAxpert photospectrometer (Qiagen). Subsequently, DNA was normalized to 10 ng/µl using the QIAgility liquid handling station (Qiagen). PCR amplification of the fungal ribosomal internal transcribed spacer region ITS2 was performed using 40 ng of template DNA and 0.4 µM of each primer, ITS3ngs (CANCGATGAAGAACGYRG) and ITS4ngs (CCTSCSCTTANTDATATGC) for fungi [11], with 1X of GoTaq® Colorless Master Mix from Promega (Promega, Madison, WI, United States), the final volume amounting to 25 µl. The PCR protocol included polymerase activation at 95°C for 5 min, followed by 35 cycles of denaturation at 95°C for 40 s, annealing at 58°C for 40 s, and elongation at 72°C for 1 min. For each sample, PCR was carried out in technical triplicates and were pooled prior to sequencing. PCR products were sent to the Functional Genomics Center Zurich (Zurich, Switzerland), for indexing followed by purification, quantification, equimolar pooling. The library was pre-sequenced on an Illumina MiniSeq platform (Illumina, San Diego, CA, United States) to inform re-pooling of samples based on read counts to obtain more even sequence distribution across samples. The re-pooled library was sequenced on a Illumina MiSeq platform using the v3 chemistry for PE300 reads. The bioinformatic analysis was based on VSEARCH [12], as described in [13]. Briefly, PhiX DNA was removed from the dataset using bowtie2 [14] and primers were trimmed using cutadapt [15]. Forward and reverse reads were merged using fastq_mergepairs, allowing staggered constructs via fastq_allowmergestagger and with the minimal overlap length set to 30. Quality stats were inspected using fastq_eestats. The fastq_filter function [16] was used to remove low-quality reads with the maximum expected error set to one. Sequence dereplication and delineation into amplicon sequence variants (ASVs) was performed using the derep_fullength and cluster_unoise [17] functions, setting minsize=8 for the latter, and subsequently deleting chimeric sequences using UCHIME2 [18]. ITSx was used to detect and extract correctly delineated ITS2 sequences [19]. The sequences remaining after quality filtering were mapped against the verified ASV sequences via usearch_global, setting identity to 0.97, maxhits to 1 and maxaccepts to 0. ASVs were taxonomically classified using the SINTAX algorithm [20] against the Unite v.83 database [21], using a cutoff of 0.8. Entries that were not assigned at the domain level or contained ambiguous base calls were excluded from the Unite database prior to taxonomic assignment. Iterative subsampling (ISS) was used to normalize the number of reads across samples, using 100 iterations and subsequently computing the mean of all iterations. The resulting average sampling-observation matrix was used to compute weighted % ASVs of AMF and plant pathogenic fungi using information from FunGUILD [22] about probable ecological guild-membership of the taxa. Similarly, % of so-called iconic fungi were computed, including the often particularly colorful grassland macrofungi of high conservational value [23] known as CHEGD taxa (encompassing the Clavariaceae, Hygrophoraceae, Entolomataceae, Geoglossaceae and Dermoloma taxa, Caboň et al., 2021).

Measuring ES indicators: Sampling August and September 2020

Soil sampling design for root mass and soil compaction: Samples were taken at three locations per plot, each 8 m apart. At each location, 5 cm × 5 cm cylindrical soil cores were taken at 0-5 cm and 5-10 cm soil depth, including organic top layer, two soil cores per depth and location. The three cores of the respective depth were pooled for further analysis.

Root mass: Root mass was used as a measure of topsoil stability and belowground productivity. For that purpose, roots were washed, dried at 105 °C for 24 h, and weighed. For statistical analysis, root biomass from 0-5 cm was used as a proxy for erosion resistance of the topsoil.

Soil compaction: Soil compaction is a direct driver of water infiltration capacity [25]. To determine bulk density the fine soil stock (FSS) was calculated according to Poeplau et al. (2017) as FSS = mass fine soil / volume soil. For this, soil cores from 5 – 10 cm depth were weighed after drying at 105 °C for 24 h. Mass fine soil was determined by subtracting the weight of roots and rocks from the soil cores used for the root analysis from the weight of the whole sample for FSS and used together with clay content to calculate packing density [27] as a measure for soil compaction.

Sampling design for soil surface P: 10 soil samples were taken per plot along a 20-m transect with a spoon resulting in topsoil samples to 1.5 cm depth. The 10 samples per plot were pooled for further analysis.

Surface P: For surface soil phosphorus (P) concentrations, a measure of eutrophication risk for freshwater ecosystems, samples were sieved to 2 mm and dried at air temperature. Water-extractable soil phosphorus (1:10 extraction) was measured photometrically (Evolution 220 with Cetac ASX-520, Thermo Fisher Scientific, Waltham, MA, United States).

Measuring ES indicators: Sampling May to mid-June 2021

Plant species richness: all vascular plant species occurring at two 2 m × 2 m quadrats were recorded and summed for a total richness in plant species.

Edible plants: Collecting edible plants for preparing, e.g., tea or salad in Switzerland depicts rather a cultural ecosystem service than a provisioning services as it might be in other cultural contexts countries [28–30]. The number of edible plant species was calculated based on the species cover information from the vegetation survey and literature information about plants considered edible [31–33].

Nectar provision: We used potential nectar provision based on flower traits as an indicator for the Pollination ES [34]. Potential nectar provision in kg ha^{-1} was estimated using the cover of plant species from the vegetation surveys and data on nectar provision per species and cover from the literature. First, data from the plant trait database [35] was used to assess whether a species provides nectar or not, then information about nectar provision per area was extracted for each of these species using data from Baude et al. (2016) and Filipiak et al. (2022). For the species for which no data were available from the latter two sources, but were indicated as species providing nectar in BiolFlor, data gaps were filled with the median for nectar provision of all the species in our study. Nectar provision of occasionally occurring tree species was set to zero, because grassland management did not allow woody species to reach a flowering stage. With the species-abundance and nectar provision data, nectar provision per plot was calculated.

Weeds: The number of agricultural weed plants (or of dense patches for clonal plants) was recorded along two 2 m × 20 m strips per site. Following species were considered relevant weeds: *Rumex obtusifolius, Colchicum spp., Senecio jacobaea, Rhinanthus spp.*, *Carlina spp.*, *Cirsium spp.*, *Anthriscus sylvestris*, and *Heracleum shpondylium*.

Sampling design for herbivory: Along two 20-m transects, leaves of one legume, one grass, and one forb (if present within a radius of 0.2 m) were gathered randomly ever 0.5 m, resulting in 80 leaves per plant functional group per plot.

Herbivory: Leaf damage by herbivorous arthropods was assessed by sampling leaves in the field and subsequent visual examination of damage. For every leaf, damage by insects was assessed (yes/no). Information about cover of legumes, grasses, and forbs from the vegetation survey was used to subsequently compute total % damaged leaves for each plot. As herbivory increased with time of sampling, we used a linear regression (% leaves damaged against day of year and grassland type, i.e., combination of meadow/pasture and Eco-scheme yes/no management) to correct herbivory for sampling date within each grassland type. An interaction term was excluded from the model, as the respective models showed no significant difference.

Sampling design for plant biomass and digestibility: Aboveground plant biomass was sampled repeatedly on the plots. In pastures, grazing exclusion cages of 1.25 m × 1.25 m were installed prior to grazing, and respective biomass was sampled by cutting four 50 cm × 50 cm quadrats at 1 cm above the surface. Samples were dried at 60 °C and weighed.

Digestibility: The biomass sample taken closest to the first date of grassland use as indicated by the farmers in a management survey were analyzed for digestibility (i.e., digestible organic matter) via enzymatic digestion in rumen fluid according to Tilley and Terry (1963).

Biomass yield: For aboveground biomass yield, vegetation biomass was sampled close to a reference date set to end of May (day of year, DOY, 146). As some plots were sampled later or earlier, either due to displacement of the grazing exclusion cages by cow activity or logistic reasons, biomass was corrected for sampling date. To this end, biomass weight was divided by the temperature-degree sum until sampling date following the approach described in Menzi et al. (1991). Modelled daily mean temperatures at 2 m above ground in a 1 km × 1 km resolution [40] were extracted in QGIS 2.18.14 for each sampling plot and then averaged over sampling plots per day to compute an average temperature sum per day of year.

Sampling design for N_2 fixation: On each plot, ten individuals of all legume species with a cover of > 0.5 % in the vegetation survey were collected within the grassland and pooled per species and plot for further analysis.

N_2 fixation: Symbiotic N fixation in biomass harvested close to DOY 146 was calculated based on the productivity measure described before (biomass yield) and by considering identity and cover of occurring legume species. The legume samples were dried at 60 °C, milled to fine powder, and analyzed for total nitrogen content (N%) using an elemental analyzer (Euro EA, HEKAtech, Wegberg). As N content was influenced by sampling date, it was corrected for day of year of sampling using a linear regression, as described before for values of leaf herbivore damage. Legume species mass percent of aboveground biomass per plot (Leg%) was calculated using a linear regression modelling the relationship between biomass percent and estimated cover of legumes on eleven plots using the vegetation within a 0.5 m × 0.5 m quadrat. To calculate symbiotic N fixation per plot (index N_{fix}), for all occurring legume species their mass percent (Leg %) was multiplied with the nitrogen content (N %) measured for the respective species i. All products were then summed and multiplied with the biomass measured on the respective plot corrected for the temperature sum (TSMenzi) as described before, according to **equation S1**.

Equation S1: $$Index\ N_{fix} = \frac{Biomass}{TS_{Menzi}} * \sum_{i=1}^{Nr.\ Leg.\ Sp.} Leg\ \%_i\ * N\ \%_i$$

Sampling design for Earthworms: three 20 cm × 20 cm × 30 cm soil pits were dug out along a 20 m-transect.

Earthworms: Earthworms can contribute to regulating the hydrological cycle via their tunneling activities and positive effects on soil aggregate formation [41]. To assess earthworm abundances, the excavated material was checked manually for earthworms. the number of all live individuals was recorded and the mean value across the three sample locations was used for further analysis. Earthworm numbers were corrected for soil moisture, which varied during the sampling period according to weather conditions, using a linear regression, as previously described for leaf herbivore damage but correcting for soil moisture instead of DOY. Soil moisture was recorded the same day as earthworm abundance with a HH2 moisture meter (Delta-T devices, Cambridge, UK) as an average of eight single values taken at the corners of the two vegetation surveys described above.

Aesthetics: We used a questionnaire based approach to assess aesthetic appreciation of the parcels [42,43]. Aesthetic appreciation of the plant community was derived from standardized pictures of each plot taken prior to the vegetation surveys. An online questionnaire survey asking people for their personal perception of the aesthetic quality of the respective grassland plant community on a 5-point Likert scale from attractive to unattractive was set up with QuestionPro (QuestionPro Inc, Austin, TX, United States), with ten of the 88 photographs randomly chosen per questionnaire. Pictures did not include any landscape feature, structure, or grazing animal but only a close up of the plant community in an area of approx. 2 m × 2 m at the day of sampling. The questionnaire was set up in German language and was online from the 11th of January to the 17th of January 2022. It was distributed via different pathways, first via e-mail to professional stakeholders in agriculture, environmental protection, tourism, and/or botanical gardens in Switzerland, and second via Twitter posts. In total, 414 participants completed the questionnaire, yielding, on average, 55 ratings per grassland picture. The average rating value per plot was computed and used as an indicator for aesthetic appreciation.

N_2O emissions: N_2O emissions per ha were calculated according to the IPCC guidelines [44], equation 11.1, using fertilizer data from the management interviews and Switzerland-specific information on livestock from Richner et al. (2017) to estimate the amount of N excreted by grazing animals. Emission factors for wet climates were used.

N leaching: Potential nitrate leaching was calculated using a tool accounting for fertilizer N and animal excreta as sources for nitrate leaching [46] with equations for grassland dairy systems. Therein, equation 12, which calculates the N leaching from applied fertilizer for baseline scenario, synthetic as well as available N from organic fertilizers was used. Soil type was set to loam, and the intermediate climate region was selected. Available nitrogen in the organic fertilizer was computed from the management information based on data from Richner et al. (2017).

Supplementary References

1. Stahel, W. *Statistische Datenanalyse*. (Vieweg und Sohn, Braunschweig, 2002).
2. Haines-Young, R. & Potschin, M. CICES V5. 1. Guidance on the application of the revised structure. *Fabis Consult.* 53 (2018).
3. Davison, A. C. & Hinkley, D. V. *Bootstrap Methods and Their Application*. (Cambridge University Press, Cambridge, 1997). doi:https://doi.org/10.1017/CBO9780511802843.
4. Niku, J., Hui, F. K. C., Taskinen, S. & Warton, D. I. gllvm: Fast analysis of multivariate abundance data with generalized linear latent variable models in r. *Methods Ecol. Evol.* **10**, 2173–2182 (2019).
5. van Buuren, S. & Groothuis-Oudshoorn, K. mice: Multivariate Imputation by Chained Equations in R. *J. Stat. Softw.* **45**, 1–67 (2011).
6. GELAN. Agrarinformationssystem GELAN, Amt für Landwirtschaft und Natur. Zollikofen, Switzerland. at http://www.gelan.ch/de/ (2019).
7. Hoop, D. & Schmid, D. *Betriebstypologie ZA-2015 Der Zentralen Auswertung von Buchhaltungsdaten. Version 1.5., Agroscope Ettenhausen*. https://ira.agroscope.ch/de-CH/publication/41862 (2020).
8. Walkley, A. & Black, I. A. An examination of the degtjareff method for determining soil organic matter, and a proposed modification of the chromic acid titration method. *Soil Sci.* **37**, 29–38 (1934).
9. Schinner, F. & Sonnleitner, R. Methoden der Bodenmikrobiologie und -biochemie. in *Bodenökologie: Mikrobiologie und Bodenenzymatik Band I* 237–259 (1996). doi:10.1007/978-3-642-80175-4_4.
10. Vance, E. D., Brookes, P. C. & Jenkinson, D. S. An extraction method for measuring soil microbial biomass C. *Soil Biol. Biochem.* **19**, 703–707 (1987).
11. Tedersoo, L. & Lindahl, B. Fungal identification biases in microbiome projects. *Environ. Microbiol. Rep.* **8**, 774–779 (2016).
12. Rognes, T., Flouri, T., Nichols, B., Quince, C. & Mahé, F. VSEARCH: A versatile open source tool for metagenomics. *PeerJ* **4**, (2016).
13. Longepierre, M. *et al.* Limited resilience of the soil microbiome to mechanical compaction within four growing seasons of agricultural management. *ISME Commun.* **1**, 1–13 (2021).
14. Langmead, B. & Salzberg, S. L. Fast gapped-read alignment with Bowtie 2. *Nat. Methods* **9**, 357–359 (2012).
15. Martin, M. Cutadapt removes adapter sequences from high-throughput sequencing reads. *Adv. Environ. Biol.* **17**, 10–12 (2011).
16. Edgar, R. C. & Flyvbjerg, H. Error filtering, pair assembly and error correction for next-generation sequencing reads. *Bioinformatics* **31**, 3476–3482 (2015).
17. Edgar, R. C. UNOISE2: improved error-correction for Illumina 16S and ITS amplicon sequencing. *bioRxiv [Preprint]* 081257 (2016).
18. Edgar, R. C. UCHIME2: improved chimera prediction for amplicon sequencing. *bioRxiv [Preprint]* 074252 (2016).
19. Bengtsson-Palme, J. *et al.* Improved software detection and extraction of ITS1 and ITS2 from

ribosomal ITS sequences of fungi and other eukaryotes for analysis of environmental sequencing data. *Methods Ecol. Evol.* **4**, 914–919 (2013).

20. Edgar, R. SINTAX: a simple non-Bayesian taxonomy classifier for 16S and ITS sequences. *bioRxiv [Preprint]* 074161 (2016).

21. Kõljalg, U. *et al.* UNITE a database providing web-based methods for the molecular identification of ectomycorrhizal fungi. *New Phytol.* **166**, 1063–1068 (2005).

22. Nguyen, N. H. *et al.* FUNGuild: An open annotation tool for parsing fungal community datasets by ecological guild. *Fungal Ecol.* **20**, 241–248 (2016).

23. Griffith, G. *et al.* The international conservation importance of Welsh 'waxcap' grasslands. *Mycosphere* **4**, 969–984 (2013).

24. Caboň, M. *et al.* Mulching has negative impact on fungal and plant diversity in Slovak oligotrophic grasslands. *Basic Appl. Ecol.* **52**, 24–37 (2021).

25. Leitinger, G., Tasser, E., Newesely, C., Obojes, N. & Tappeiner, U. Seasonal dynamics of surface runoff in mountain grassland ecosystems differing in land use. *J. Hydrol.* **385**, 95–104 (2010).

26. Poeplau, C., Vos, C. & Don, A. Soil organic carbon stocks are systematically overestimated by misuse of the parameters bulk density and stone content. *SOIL Discuss.* **3**, 61–66 (2017).

27. Renger, M. Über den Einfluss der Dränung auf das Gefüge und die Wasserdurchlässigkeit bindiger Böden. *Mitteilungen Dtsch. Bodenkundliche Gesellschaft* **11**, 23–28 (1970).

28. Abbet, C. *et al.* Ethnobotanical survey on wild alpine food plants in Lower and Central Valais (Switzerland). *J. Ethnopharmacol.* **151**, 624–634 (2014).

29. Tardío, J., Pardo-De-Santayana, M. & Morales, R. Ethnobotanical review of wild edible plants in Spain. *Bot. J. Linn. Soc.* **152**, 27–71 (2006).

30. Schulp, C. J. E., Thuiller, W. & Verburg, P. H. Wild food in Europe: A synthesis of knowledge and data of terrestrial wild food as an ecosystem service. *Ecol. Econ.* **105**, 292–305 (2014).

31. Pfister, T. & auf der Mauer, F. *Aromatische Bergkräuter Für Die Naturküche Sammeln Und Zubereiten*. (Haupt verlag, 2017).

32. Machatschek, M. & Mautner, E. *Speisekammer Aus Der Natur Bevorratung Und Haltbarmachung von Wildpflanzen*. (Böhlau Verlag, 2015).

33. Höller, A. & Grappendorf, D. *Essbare Wildsamen Finden, Sammeln Und Geniessen*. (Ulmer Verlag, 2019).

34. Bartholomée, O. & Lavorel, S. Disentangling the diversity of definitions for the pollination ecosystem service and associated estimation methods. *Ecol. Indic.* **107**, 105576 (2019).

35. Kühn, I., Durka, W. & Klotz, S. BiolFlor : A new plant-trait database as a tool for plant invasion ecology. *Divers. Distrib.* **10**, 363–365 (2004).

36. Baude, M. *et al.* Historical nectar assessment reveals the fall and rise of floral resources in Britain. *Nature* **530**, 85–88 (2016).

37. Filipiak, M., Walczyńska, A., Denisow, B., Petanidou, T. & Ziółkowska, E. Phenology and production of pollen, nectar, and sugar in 1612 plant species from various environments. *Ecology* **103**, 2021–2022 (2022).

38. Tilley, J. M. A. & Terry, R. A. A two-stage technique for the in vitro digestion of forage crops. *Grass Forage Sci.* **18**, 104–111 (1963).

39. Menzi, H., Blum, H. & Nössberger, J. Relationship between climatic factors and the dry matter production of swards of different composition at two altitudes. *Grass Forage Sci.* **46**, 223–230 (1991).

40. Federal Office of Meteorology and Climatology MeteoSwiss. *Documentation of MeteoSwiss Grid-Data Products: Daily Mean, Minimum and Maximum Temperature: TabsD, TminD, TmaxD*. (2021).

41. Keith, A. M. & Robinson, D. a. Earthworms as natural capital : ecosystem service providers in agricultural soils. *Econ. J.* **II**, 91–99 (2012).

42. Lindemann-Matthies, P., Briegel, R., Schüpbach, B. & Junge, X. Aesthetic preference for a Swiss alpine landscape: The impact of different agricultural land-use with different biodiversity. *Landsc. Urban Plan.* **98**, 99–109 (2010).

43. Zoderer, B. M., Tasser, E., Erb, K. H., Lupo Stanghellini, P. S. & Tappeiner, U. Identifying and mapping the tourists' perception of cultural ecosystem services: A case study from an Alpine region. *Land use policy* **56**, 251–261 (2016).

44. IPCC. N2O emissions from managed soils, and CO2 emissions from lime and urea application. in *2019 Refinement to the 2006 IPCC Guidelines for National Greenhouse Gas Inventories* (2019).

45. Richner, W., Flisch, R., Mayer, J. & Schlegel, P. 4 / Eigenschaften und Anwendung von Düngern. **8**, 1–24 (2017).

46. Martin, I., Davison, P. & Bramer, S. *The Nitrate Leaching Tool - Technical Reference*. (2021).

Supplementary Code

R-code for data preparation

```
# loading Data
all_ES <- read.table("RawData.txt", sep="", header=T)

# ____________________________________________________________
# ____________________________________________________________

# 1) ------- NA replacement ------------------------------------------
library(mice)

d.data <- all_ES[, -c(26)] # deleting plot number, as not needed here
d.data$Prod_system <- as.factor(d.data$Prod_system)
d.data$Eco_scheme <- as.factor(d.data$Eco_scheme)
d.data$Harv_type <- as.factor(d.data$Harv_type)

rownames(d.data) <- 1:nrow(d.data)

imp <- mice(data=d.data, m=30, maxit=10, seed=29976, print=FALSE)

#Extract imputed values because "with()" does not run with gllvm() objects

#Biomass
imp.biomass <- as.matrix(imp$imp$Biomass)
d.data$Biomass[c(2, 5, 20, 39, 65)] <- apply(imp.biomass, 1, median)

#Digestibility
imp.digest <- as.numeric(imp$imp$Digestibility)
d.data$Digestibility[20] <- median(imp.digest)
```

```
#Aesthetics
imp.aesthet <- as.numeric(imp$imp$Aesthetics)
d.data$Aesthetics[23] <- median(imp.aesthet)


# complete N_fix_log -> necessary because Biomass (which was NA-filled) is part of the calculation for N_fix
colnames(all_ES)
d.data_wNfix <- cbind(d.data, all_ES[, 18])
colnames(d.data_wNfix)[26] <- "N_fix_RAW"
d.data_wNfix[2,26] <- 0.001131272 * d.data_wNfix[2,1]
d.data_wNfix[5,26] <- 0.00119149730440437 * d.data_wNfix[5,1]
d.data_wNfix[20,26] <- 0.002897494 * d.data_wNfix[20,1]
d.data_wNfix[39,26] <- 0.003873794 * d.data_wNfix[39,1]
d.data_wNfix[65,26] <- 0.000677954 * d.data_wNfix[65,1]


d.data_wNfix2 <- d.data_wNfix[, c(1:9, 26; 11:25)]


# ____________________________________________________________________________
# ____________________________________________________________________________


# 2) Data distribution: log transformation and zero replacement if necessary


all_ES <- d.data_wNfix2


# P
all_ES$logP <- log(all_ES$P)


# N leach
all_ES$logN_leach <- log(all_ES$N_leach)


# Weeds
```

```
all_ES$log_weeds <- log(all_ES$Weeds)
quant.1 <- quantile( all_ES$Weeds[all_ES$Weeds!=0] )
c.1 <- as.numeric( quant.1[2]^2 / quant.1[4] )
all_ES$Weeds.t <- all_ES$Weeds + c.1
all_ES$log_weeds <- log(all_ES$Weeds.t)

# N2O
all_ES$log_N2O <- log(all_ES$N2O)
quant.2 <- quantile( all_ES$N2O[all_ES$N2O!=0] )
c.2 <- as.numeric( quant.2[2]^2 / quant.2[4] )
all_ES$N2O.t <- all_ES$N2O + c.2
all_ES$log_N2O <- log(all_ES$N2O.t)

# Iconic_fungi
all_ES$log_Iconic_fungi <- log(all_ES$Iconic_fungi)

# Cow_days
all_ES$log_cow_days <- log(all_ES$Cow_days)
quant.3 <- quantile( all_ES$Cow_days[all_ES$Cow_days!=0] )
c.3 <- as.numeric( quant.3[2] / quant.3[4] )
all_ES$Cow_days.t <- all_ES$Cow_days + c.3
all_ES$log_cow_days <- log(all_ES$Cow_days.t)

# N2 fixation
all_ES$log_N_fix <- log(all_ES$N_fix)

# Nectar
all_ES$log_Nectar <- log(all_ES$Nectar)

# Edible plants
all_ES$log_Edible_plants <- log(all_ES$Edible_plants)
```

```
# ____________________________________________________________________
# ____________________________________________________________________

# 3) ------- shifting log transformed variables with negative values to positive range ------

# P
min(all_ES$logP)
all_ES$logP <- all_ES$logP + min(all_ES$logP)*-1

# Weeds
min(all_ES$log_weeds)
all_ES$log_weeds <- all_ES$log_weeds + min(all_ES$log_weeds)*-1

# N2O
min(all_ES$log_N2O)
all_ES$log_N2O <- all_ES$log_N2O + min(all_ES$log_N2O)*-1

# Iconic fungi
min(all_ES$log_Iconic_fungi)
all_ES$log_Iconic_fungi <- all_ES$log_Iconic_fungi + min(all_ES$log_Iconic_fungi)*-1

# Cow days
min(all_ES$log_cow_days)
all_ES$log_cow_days <- all_ES$log_cow_days + min(all_ES$log_cow_days)*-1

# N fix
min(all_ES$log_N_fix)
all_ES$log_N_fix <- all_ES$log_N_fix + min(all_ES$log_N_fix)*-1


# ____________________________________________________________________
# ____________________________________________________________________
```

```
# 4) ------- dividing by Max ------------------------------------

# Order dataset

all_ES2 <- all_ES[, c(1, 2, 3, 4, 36, 6, 7, 8, 28, 35, 11, 12, 13, 14, 27, 26, 30, 18, 37, 32, 33, 22, 23:25)]
all_ES <- all_ES2

a <- all_ES[,1]/max(all_ES[, 1])
b <- all_ES[,2]/max(all_ES[, 2])
c <- all_ES[,3]/max(all_ES[, 3])
d <- all_ES[,4]/max(all_ES[, 4])
e <- all_ES[,5]/max(all_ES[, 5])
f <- all_ES[,6]/max(all_ES[, 6])
g <- all_ES[,7]/max(all_ES[, 7])
h <- all_ES[,8]/max(all_ES[, 8])
i <- all_ES[,9]/max(all_ES[, 9])
j <- all_ES[,10]/max(all_ES[, 10])
k <- all_ES[,11]/max(all_ES[, 11])
l <- all_ES[,12]/max(all_ES[, 12])
m <- all_ES[,13]/max(all_ES[, 13])
n <- all_ES[,14]/max(all_ES[, 14])
o <- all_ES[,15]/max(all_ES[, 15])
p <- all_ES[,16]/max(all_ES[, 16])
q <- all_ES[,17]/max(all_ES[, 17])
r <- all_ES[,18]/max(all_ES[, 18])
s <- all_ES[,19]/max(all_ES[, 19])
t <- all_ES[,20]/max(all_ES[, 20])
u <- all_ES[,21]/max(all_ES[, 21])
v <- all_ES[,22]/max(all_ES[, 22])

all_ES_max <- cbind(a, b, c, d, e, f, g, h, i, j, k, l, m, n, o, p, q, r, s, t, u, v, all_ES[, 23:25])
```

```
# Names
colnames(all_ES_max) <- colnames(all_ES)

# ________________________________________________________________
# ________________________________________________________________

# 5) ------- Reversing values for Dis-Services ------------------

all_ES_max[, 16] <- max(na.omit(all_ES_max[, 16])) - all_ES_max[, 16]
colnames(all_ES_max)[16] <- "less_P_log"
all_ES_max[, 15] <- max(na.omit(all_ES_max[, 15])) - all_ES_max[, 15]
colnames(all_ES_max)[15] <- "less_N_leach_log"
all_ES_max[, 9] <- max(na.omit(all_ES_max[, 9])) - all_ES_max[, 9]
colnames(all_ES_max)[9] <- "less_Weeds_log"
all_ES_max[, 17] <- max(na.omit(all_ES_max[, 17])) - all_ES_max[, 17]
colnames(all_ES_max)[17] <- "less_N2O_log"
all_ES_max[, 13] <- max(na.omit(all_ES_max[, 13])) - all_ES_max[, 13]
colnames(all_ES_max)[13] <- "less_Heavy_metals"
all_ES_max[, 4] <- max(na.omit(all_ES_max[, 4])) - all_ES_max[, 4]
colnames(all_ES_max)[4] <- "less_soil_compac"
all_ES_max[, 7] <- max(na.omit(all_ES_max[, 7])) - all_ES_max[, 7]
colnames(all_ES_max)[7] <- "less_Plant_path"
all_ES_max[, 8] <- max(na.omit(all_ES_max[, 8])) - all_ES_max[, 8]
colnames(all_ES_max)[8] <- "less_Herbivory"

all_ES_max <- as.data.frame(all_ES_max)

colnames(all_ES_max)[5]<- "Nectar_log"
colnames(all_ES_max)[10]<- "N_fix_log"
colnames(all_ES_max)[19]<- "Edible_plants_log"
colnames(all_ES_max)[20]<- "Iconic_fungi_log"
```

```
colnames(all_ES_max)[21]<- "Cow_days_log"
```

R-code for calculating GLLVM

```
library(gllvm)

# loading Data

d.data <- read.table("Transformed_for_GLLVM_Fig2_3_4.txt", header=T, sep="")

pairs <- read.table("Pairs_and_environment.txt", header=T, sep="")
d.data$Farmpair <- pairs$Farm_Pair

d.data$pH <- scale(pairs$pH)
d.data$elevation <- scale(pairs$elevation)
d.data$inclination <- scale(pairs$inclination)
d.data$northness <- scale(pairs$northness)
d.data$sand <- scale(pairs$sand)

d.data$Prod_system <- as.factor(d.data$Prod_system)
d.data$Harv_type <- as.factor(d.data$Harv_type)
d.data$Eco_scheme <- as.factor(d.data$Eco_scheme)
d.data$Farmpair <- as.factor(d.data$Farmpair)

all_ES <- d.data[, 1:22]
env <- d.pairs[, 23:32]

all_ES <- all_ES[,c(22:1)]

# Models
```

```
# Model a

fita <- gllvm(all_ES, env , formula = ~ Prod_system + Eco_scheme + Harv_type,
row.eff=~(1|Farmpair), family = "gaussian", num.lv=2, method="LA")

AICc(fita)


# Model b

fitb <- gllvm(all_ES, env , formula = ~ Prod_system + Eco_scheme + Harv_type + pH +sand +
elevation, row.eff=~(1|Farmpair), family = "gaussian", num.lv=2, method="LA")

AICc(fitb)


# Model c

fitc <- gllvm(all_ES, env , formula = ~ Prod_system + Eco_scheme + Harv_type + pH + sand + elevation
+ Prod_system:Eco_scheme, row.eff=~(1|Farmpair), family = "gaussian", num.lv=2, method="LA")

AICc(fitc)


# Model d

fitd <- gllvm(all_ES, env , formula = ~ Prod_system + Eco_scheme + Harv_type + pH + sand + elevation
+ Prod_system:Harv_type, row.eff=~(1|Farmpair), family = "gaussian", num.lv=2, method="LA")

AICc(fitd)


# Model e

fite <- gllvm(all_ES, env , formula = ~ Prod_system + Eco_scheme + Harv_type + pH + sand + elevation
+ Eco_scheme:Harv_type, row.eff=~(1|Farmpair), family = "gaussian", num.lv=2, method="LA")

AICc(fite)


# Model 1

fit1 <- gllvm(all_ES, env, formula = ~ Prod_system + Eco_scheme + Harv_type + pH + sand + elevation
+ northness + inclination, row.eff=~(1|Farmpair), family = "gaussian", num.lv=2, method="LA")

AICc(fit1)


# Model 2

fit2 <- gllvm(all_ES, env, formula = ~ Prod_system + Eco_scheme + Harv_type +
Prod_system:Eco_scheme, row.eff=~(1|Farmpair), family = "gaussian", num.lv=2, method="LA")
```

```
AICc(fit2)


# Model 3

fit3 <- gllvm(all_ES, env, formula = ~ Prod_system + Eco_scheme + Harv_type +
Prod_system:Harv_type, row.eff=~(1|Farmpair), family = "gaussian", num.lv=2, method="LA")

AICc(fit3)


# Model 4

fit4 <- gllvm(all_ES, env, formula = ~ Prod_system + Eco_scheme + Harv_type +
Eco_scheme:Harv_type, row.eff=~(1|Farmpair), family = "gaussian", num.lv=2, method="LA")

AICc(fit4)
```

R-code for SEM

```
library(lavaan)

library(AICcmodavg)

library(lavaanPlot)


#### 1 Experimental design


design.myPlots <- read.table("Pairs_and_environment.txt", sep="", header=T)

# ______________________________________________________________

# ______________________________________________________________


# grassland, topo, and management


design.myPlots$DEM_scaled <- design.myPlots$elevation / 100

design.myPlots$N_av_scaled <- design.myPlots$N_average / 10

hist(design.myPlots$N_av_scaled)

design.myPlots$Graz_av_scaled <- design.myPlots$Graz_average / 100

hist(design.myPlots$Graz_av_scaled)
```

```
ES1 <- read.table("Transformed_for_GLLVM_Fig2_3_4.txt", sep="", header=T)
all <- cbind(ES1, design.myPlots)
colnames(all)
all$Manage2 <-all$Harv_type
all$Intens2 <-all$Eco_scheme
all$System2 <-all$Prod_sytem
all$Inklination21 <- all$inclination
all$Northness2 <- all$northness


# first, correlating management vars:
library(ggpubr)
library(ggplot2)

N_C <-ggplot(design.myPlots, aes(x=N_average, y=Cut_average))+
  geom_point(size=3, alpha=0.3)+
  #geom_smooth(method="lm", color="grey30", alpha=0.3)+
  theme_classic(base_size=16)+
  xlab(expression(bold(Fertilizer) ~ bold(N) ~  "[kg N ha"^-1 ~ year^-1 *"]"))+
  ylab(expression(bold(Nr.) ~ bold(of) ~ bold(cuts) ~ "[year"^-1 *"]"))
#stat_cor(method = "spearman", label.x = 3, label.y = 4.2, size=4.5)

N_G <-ggplot(design.myPlots, aes(x=Graz_average, y=N_average))+
  geom_point(size=3, alpha=0.3)+
  #geom_smooth(method="lm", color="grey30", alpha=0.3)+
  theme_classic(base_size=16)+
  xlab(expression(bold(Grazing) ~ bold(intensity) ~  "[kg N ha"^-1 ~ year^-1 *"]"))+
  ylab(expression(bold(Fertilizer) ~ bold(N) ~ "[LU days ha"^-1 ~ year^-1 *"]"))
#stat_cor(method = "spearman", label.x = 3, label.y = 250, size=4.5)

G_C <-ggplot(design.myPlots, aes(x=Graz_average, y=Cut_average))+
```

```
  geom_point(size=3, alpha=0.3)+
  #geom_smooth(method="lm", color="grey30", alpha=0.3)+
  theme_classic(base_size=16)+
  xlab(expression(bold(Grazing) ~ bold(intensity) ~ "[LU days ha"^-1 ~ year^-1 *"]"))+
  ylab(expression(bold(Nr.) ~ bold(of) ~ bold(cuts) ~ "[year"^-1 *"]"))
#stat_cor(method = "spearman", label.x = 3, label.y = 4.2, size=4.5)

ggarrange(N_C, N_G, G_C, ncol=3, nrow=1)



# 1) with nothing transformed
BMFW <- "
Manage2 ~   DEM25  + Inklination21 + Northness2
Intens2 ~   DEM25  + Inklination21 + Northness2

"
semBMFW <- sem(BMFW,  dat=all )  #
varTable(semBMFW)
summary(semBMFW, fit.measures = TRUE, rsq = TRUE, standardized=TRUE) ###

# 2) with  DEM scaled
BMFW <- "
Manage2 ~   DEM_scaled  + Inklination21 + Northness2
Intens2 ~   DEM_scaled  + Inklination21 + Northness2
"
semBMFW <- sem(BMFW,  dat=all )  # no warning message
summary(semBMFW, fit.measures = TRUE, rsq = TRUE, standardized=TRUE) ### model estimates and fit indices are exactly the same

# --> As only Inclination has sign effects, that is the only one we keep for the further models.
```

```
# final Model:

BMFW <- "
Manage2 ~   Inklination21
Intens2 ~    Inklination21

N_av_scaled  ~ System2 + Manage2 + Intens2
Graz_av_scaled ~ System2 + Manage2 + Intens2
Cut_average ~ System2 + Manage2 + Intens2

N_av_scaled  ~~ Graz_av_scaled
Graz_av_scaled  ~~ Cut_average
Cut_average ~~ N_av_scaled
"
semBMFW <- sem(BMFW,  dat=all )  # no warning message
summary(semBMFW, fit.measures = TRUE, rsq = TRUE, standardized=TRUE) ###

mylist <- list()

# 1) Biomass:

BMFW <- "
Manage2 ~   Inklination21
Intens2 ~    Inklination21

N_av_scaled  ~ System2 + Manage2 + Intens2
Graz_av_scaled ~ System2 + Manage2 + Intens2
Cut_average ~ System2 + Manage2 + Intens2

Biomass ~ N_av_scaled + Graz_av_scaled + Cut_average + Manage2 + Intens2 + System2

N_av_scaled  ~~ Graz_av_scaled
```

```
Graz_av_scaled  ~~ Cut_average
Cut_average ~~ N_av_scaled
"
semBMFW <- sem(BMFW,  dat=all )  # no warning message
summary(semBMFW, fit.measures = TRUE, rsq = TRUE, standardized=TRUE) ###
summary(semBMFW, standardized=T)$pe[c(1,3,10)]



mylist[[1]] <- summary(semBMFW, standardized=T)$pe$std.all


# 2) Digestibility:


BMFW <- "
Manage2 ~   Inklination21
Intens2 ~    Inklination21


N_av_scaled  ~ System2 + Manage2 + Intens2
Graz_av_scaled ~ System2 + Manage2 + Intens2
Cut_average ~ System2 + Manage2 + Intens2


Digestibility ~ N_av_scaled + Graz_av_scaled + Cut_average + Manage2 + Intens2 + System2


N_av_scaled  ~~ Graz_av_scaled
Graz_av_scaled  ~~ Cut_average
Cut_average ~~ N_av_scaled
"
semBMFW <- sem(BMFW,  dat=all )  # warning message due to digestibility having low variances...
varTable(semBMFW)
summary(semBMFW, fit.measures = TRUE, rsq = TRUE, standardized=TRUE) ###
mylist[[2]] <- summary(semBMFW, standardized=T)$pe$std.all


# 3) Root mass:
```

```
BMFW <- "
Manage2 ~   Inklination21
Intens2 ~    Inklination21

N_av_scaled  ~ System2 + Manage2 + Intens2
Graz_av_scaled ~ System2 + Manage2 + Intens2
Cut_average ~ System2 + Manage2 + Intens2

Rootmass ~ N_av_scaled + Graz_av_scaled + Cut_average + Manage2 + Intens2 + System2

N_av_scaled  ~~ Graz_av_scaled
Graz_av_scaled  ~~ Cut_average
Cut_average ~~ N_av_scaled
"
semBMFW <- sem(BMFW,  dat=all )  #
varTable(semBMFW)
summary(semBMFW, fit.measures = TRUE, rsq = TRUE, standardized=TRUE) ###
mylist[[3]] <- summary(semBMFW, standardized=T)$pe$std.all

# 4) Plant richness:

BMFW <- "
Manage2 ~   Inklination21
Intens2 ~    Inklination21

N_av_scaled  ~ System2 + Manage2 + Intens2
Graz_av_scaled ~ System2 + Manage2 + Intens2
Cut_average ~ System2 + Manage2 + Intens2

Plant_richness ~ N_av_scaled + Graz_av_scaled + Cut_average + Manage2 + Intens2 + System2
```

```
N_av_scaled  ~~ Graz_av_scaled
Graz_av_scaled  ~~ Cut_average
Cut_average ~~ N_av_scaled
"
semBMFW <- sem(BMFW,  dat=all )  # warning message, plant richness has low variance
varTable(semBMFW)
summary(semBMFW, fit.measures = TRUE, rsq = TRUE, standardized=TRUE) ###
mylist[[4]] <- summary(semBMFW, standardized=T)$pe$std.all

# 5) less Plant path:

BMFW <- "
Manage2 ~   Inklination21
Intens2 ~    Inklination21

N_av_scaled  ~ System2 + Manage2 + Intens2
Graz_av_scaled ~ System2 + Manage2 + Intens2
Cut_average ~ System2 + Manage2 + Intens2

less_Plant_path ~ N_av_scaled + Graz_av_scaled + Cut_average + Manage2 + Intens2 + System2

N_av_scaled  ~~ Graz_av_scaled
Graz_av_scaled  ~~ Cut_average
Cut_average ~~ N_av_scaled
"
semBMFW <- sem(BMFW,  dat=all )  # warning message, plant path has low variance
varTable(semBMFW)
summary(semBMFW, fit.measures = TRUE, rsq = TRUE, standardized=TRUE) ###
mylist[[5]] <- summary(semBMFW, standardized=T)$pe$std.all

# 6) less Herbivory:
```

```
BMFW <- "
Manage2 ~   Inklination21
Intens2 ~    Inklination21

N_av_scaled  ~ System2 + Manage2 + Intens2
Graz_av_scaled ~ System2 + Manage2 + Intens2
Cut_average ~ System2 + Manage2 + Intens2

less_Herbivory ~ N_av_scaled + Graz_av_scaled + Cut_average + Manage2 + Intens2 + System2

N_av_scaled  ~~ Graz_av_scaled
Graz_av_scaled  ~~ Cut_average
Cut_average ~~ N_av_scaled
"
semBMFW <- sem(BMFW,  dat=all )  # warning message, plant path has low variance
varTable(semBMFW)
summary(semBMFW, fit.measures = TRUE, rsq = TRUE, standardized=TRUE) ###
mylist[[6]] <- summary(semBMFW, standardized=T)$pe$std.all



# 7) fewer Weeds:

BMFW <- "
Manage2 ~   Inklination21
Intens2 ~    Inklination21

N_av_scaled  ~ System2 + Manage2 + Intens2
Graz_av_scaled ~ System2 + Manage2 + Intens2
Cut_average ~ System2 + Manage2 + Intens2

less_Weeds_log ~ N_av_scaled + Graz_av_scaled + Cut_average + Manage2 + Intens2 + System2
```

```
N_av_scaled  ~~ Graz_av_scaled
Graz_av_scaled  ~~ Cut_average
Cut_average ~~ N_av_scaled
"
semBMFW <- sem(BMFW,  dat=all )  #
varTable(semBMFW)
summary(semBMFW, fit.measures = TRUE, rsq = TRUE, standardized=TRUE) ###
mylist[[7]] <- summary(semBMFW, standardized=T)$pe$std.all

# 8) N fix:

BMFW <- "
Manage2 ~   Inklination21
Intens2 ~    Inklination21

N_av_scaled  ~ System2 + Manage2 + Intens2
Graz_av_scaled ~ System2 + Manage2 + Intens2
Cut_average ~ System2 + Manage2 + Intens2

N_fix_log ~ N_av_scaled + Graz_av_scaled + Cut_average + Manage2 + Intens2 + System2

N_av_scaled  ~~ Graz_av_scaled
Graz_av_scaled  ~~ Cut_average
Cut_average ~~ N_av_scaled
"
semBMFW <- sem(BMFW,  dat=all )  #
varTable(semBMFW)
summary(semBMFW, fit.measures = TRUE, rsq = TRUE, standardized=TRUE) ###
mylist[[8]] <- summary(semBMFW, standardized=T)$pe$std.all

# 9) AM Fungi:
```

```
BMFW <- "
Manage2 ~   Inklination21
Intens2 ~    Inklination21

N_av_scaled  ~ System2 + Manage2 + Intens2
Graz_av_scaled ~ System2 + Manage2 + Intens2
Cut_average ~ System2 + Manage2 + Intens2

AM_Fungi ~ N_av_scaled + Graz_av_scaled + Cut_average + Manage2 + Intens2 + System2

N_av_scaled  ~~ Graz_av_scaled
Graz_av_scaled  ~~ Cut_average
Cut_average ~~ N_av_scaled
"
semBMFW <- sem(BMFW,  dat=all )  #
varTable(semBMFW)
summary(semBMFW, fit.measures = TRUE, rsq = TRUE, standardized=TRUE) ###
mylist[[9]] <- summary(semBMFW, standardized=T)$pe$std.all


# 10) Earthworms:

BMFW <- "
Manage2 ~   Inklination21
Intens2 ~    Inklination21

N_av_scaled  ~ System2 + Manage2 + Intens2
Graz_av_scaled ~ System2 + Manage2 + Intens2
Cut_average ~ System2 + Manage2 + Intens2

Earthworms ~ N_av_scaled + Graz_av_scaled + Cut_average + Manage2 + Intens2 + System2
```

```
N_av_scaled  ~~ Graz_av_scaled
Graz_av_scaled  ~~ Cut_average
Cut_average ~~ N_av_scaled
"
semBMFW <- sem(BMFW,  dat=all )  # warning message
varTable(semBMFW)
summary(semBMFW, fit.measures = TRUE, rsq = TRUE, standardized=TRUE) ###
mylist[[10]] <- summary(semBMFW, standardized=T)$pe$std.all

# 11) N leaching:


BMFW <- "
Manage2 ~   Inklination21
Intens2 ~    Inklination21


N_av_scaled  ~ System2 + Manage2 + Intens2
Graz_av_scaled ~ System2 + Manage2 + Intens2
Cut_average ~ System2 + Manage2 + Intens2

less_N_leach_log ~ N_av_scaled + Graz_av_scaled + Cut_average + Manage2 + Intens2 + System2

N_av_scaled  ~~ Graz_av_scaled
Graz_av_scaled  ~~ Cut_average
Cut_average ~~ N_av_scaled

"
semBMFW <- sem(BMFW,  dat=all )  #
varTable(semBMFW)
summary(semBMFW, fit.measures = TRUE, rsq = TRUE, standardized=TRUE) ###

mylist[[11]] <- summary(semBMFW, standardized=T)$pe$std.all
```

```
# 12) less P:

BMFW <- "
Manage2 ~   Inklination21
Intens2 ~    Inklination21

N_av_scaled  ~ System2 + Manage2 + Intens2
Graz_av_scaled ~ System2 + Manage2 + Intens2
Cut_average ~ System2 + Manage2 + Intens2

less_P_log ~ N_av_scaled + Graz_av_scaled + Cut_average + Manage2 + Intens2 + System2

N_av_scaled  ~~ Graz_av_scaled
Graz_av_scaled  ~~ Cut_average
Cut_average ~~ N_av_scaled
"
semBMFW <- sem(BMFW,  dat=all )  #
varTable(semBMFW)
summary(semBMFW, fit.measures = TRUE, rsq = TRUE, standardized=TRUE) ###
mylist[[12]] <- summary(semBMFW, standardized=T)$pe$std.all

# 13) less N2o:

BMFW <- "
Manage2 ~   Inklination21
Intens2 ~    Inklination21

N_av_scaled  ~ System2 + Manage2 + Intens2
Graz_av_scaled ~ System2 + Manage2 + Intens2
Cut_average ~ System2 + Manage2 + Intens2
```

```
less_N2O_log ~ N_av_scaled + Graz_av_scaled + Cut_average + Manage2 + Intens2 + System2

N_av_scaled  ~~ Graz_av_scaled
Graz_av_scaled  ~~ Cut_average
Cut_average ~~ N_av_scaled
"
semBMFW <- sem(BMFW,  dat=all )  #
varTable(semBMFW)
summary(semBMFW, fit.measures = TRUE, rsq = TRUE, standardized=TRUE) ###
mylist[[13]] <- summary(semBMFW, standardized=T)$pe$std.all

# 14) Edible Plants:

BMFW <- "
Manage2 ~   Inklination21
Intens2 ~    Inklination21

N_av_scaled  ~ System2 + Manage2 + Intens2
Graz_av_scaled ~ System2 + Manage2 + Intens2
Cut_average ~ System2 + Manage2 + Intens2

Edible_plants_log ~ N_av_scaled + Graz_av_scaled + Cut_average + Manage2 + Intens2 + System2

N_av_scaled  ~~ Graz_av_scaled
Graz_av_scaled  ~~ Cut_average
Cut_average ~~ N_av_scaled
"
semBMFW <- sem(BMFW,  dat=all )  # warning message
varTable(semBMFW)
summary(semBMFW, fit.measures = TRUE, rsq = TRUE, standardized=TRUE) ###
mylist[[14]] <- summary(semBMFW, standardized=T)$pe$std.all
```

```
# 15) Iconic fungi:

BMFW <- "
Manage2 ~   Inklination21
Intens2 ~    Inklination21

N_av_scaled  ~ System2 + Manage2 + Intens2
Graz_av_scaled ~ System2 + Manage2 + Intens2
Cut_average ~ System2 + Manage2 + Intens2

Iconic_fungi_log ~ N_av_scaled + Graz_av_scaled + Cut_average + Manage2 + Intens2 + System2

N_av_scaled  ~~ Graz_av_scaled
Graz_av_scaled  ~~ Cut_average
Cut_average ~~ N_av_scaled
"
semBMFW <- sem(BMFW,  dat=all )  #
varTable(semBMFW)
summary(semBMFW, fit.measures = TRUE, rsq = TRUE, standardized=TRUE) ###
mylist[[15]] <- summary(semBMFW, standardized=T)$pe$std.all

# 16) Cow days:

BMFW <- "
Manage2 ~   Inklination21
Intens2 ~    Inklination21

N_av_scaled  ~ System2 + Manage2 + Intens2
Graz_av_scaled ~ System2 + Manage2 + Intens2
Cut_average ~ System2 + Manage2 + Intens2
```

```
Cow_days_log ~ N_av_scaled + Graz_av_scaled + Cut_average + Manage2 + Intens2 + System2

N_av_scaled  ~~ Graz_av_scaled
Graz_av_scaled  ~~ Cut_average
Cut_average ~~ N_av_scaled
"
semBMFW <- sem(BMFW,  dat=all )  #
varTable(semBMFW)
summary(semBMFW, fit.measures = TRUE, rsq = TRUE, standardized=TRUE) ###
mylist[[16]] <- summary(semBMFW, standardized=T)$pe$std.all

# 17) Aesthetics:

BMFW <- "
Manage2 ~   Inklination21
Intens2 ~    Inklination21

N_av_scaled  ~ System2 + Manage2 + Intens2
Graz_av_scaled ~ System2 + Manage2 + Intens2
Cut_average ~ System2 + Manage2 + Intens2

Aesthetics ~ N_av_scaled + Graz_av_scaled + Cut_average + Manage2 + Intens2 + System2

N_av_scaled  ~~ Graz_av_scaled
Graz_av_scaled  ~~ Cut_average
Cut_average ~~ N_av_scaled
"
semBMFW <- sem(BMFW,  dat=all )  # warning message
varTable(semBMFW)
summary(semBMFW, fit.measures = TRUE, rsq = TRUE, standardized=TRUE) ###
mylist[[17]] <- summary(semBMFW, standardized=T)$pe$std.all
```

```
#lavaanPlot(model=semBMFW, node_options = list(shape = "box", fontname = "Helvetica"),
# edge_options = list(color = "grey"), coefs = TRUE,  stand=TRUE, covs = FALSE)




############ making a big dataframe with the extracted info for the plot


direct <- data.frame(matrix(nrow = 0, ncol = 3))
colnames(direct) <- c("Ext", "Bio", "We")

indirect_N <- data.frame(matrix(nrow = 0, ncol = 3))
colnames(indirect_N) <- c("Ext", "Bio", "We")

indirect_Cut <- data.frame(matrix(nrow = 0, ncol = 3))
colnames(indirect_Cut) <- c("Ext", "Bio", "We")

indirect_Graz <- data.frame(matrix(nrow = 0, ncol = 3))
colnames(indirect_Graz) <- c("Ext", "Bio", "We")



for(i in 1:17){
  direct[i,1] <- mylist[[i]][16]
  direct[i,2] <- mylist[[i]][17]
  direct[i,3] <- mylist[[i]][15]

  indirect_N[i,1] <- mylist[[i]][5] * mylist[[i]][12]
  indirect_N[i,2] <- mylist[[i]][3] * mylist[[i]][12]
  indirect_N[i,3] <- mylist[[i]][4] * mylist[[i]][12]

  indirect_Cut[i,1] <- mylist[[i]][11] * mylist[[i]][14]
  indirect_Cut[i,2] <- mylist[[i]][9] * mylist[[i]][14]
  indirect_Cut[i,3] <- mylist[[i]][10] * mylist[[i]][14]
```

```
  indirect_Graz[i,1] <- mylist[[i]][8] * mylist[[i]][13]
  indirect_Graz[i,2] <- mylist[[i]][6] * mylist[[i]][13]
  indirect_Graz[i,3] <- mylist[[i]][7] * mylist[[i]][13]
}

longer <- rbind(direct, indirect_N, indirect_Cut, indirect_Graz)
longer$categ <- rep(c("Direct", "via fertilizer N", "via Nr. of cuts", "via grazing intensity"), each=17)

library(reshape2)
even_longer <- melt(longer, id.vars=c("categ"))
even_longer$indicators <- rep(c("Biomass yield", "Digestibility", "Root mass", "Plant richness",
                    "less Plant path.", "less Herbivory", "less Weeds", "N2 fixation",
                    "AM fungi", "Earthworms", "less N leaching",
                    "less Surface P", "less N2O emissions", "Edible Plants",
                    "Iconic fungi", "Livestock presence", "Aesthetic"), 12)

#### for empty symbols if GLLVM insignificant:

#even_longer$color_var <- even_longer$variable
# Ext
even_longer$color_var <- ifelse(even_longer$variable=="Ext" & even_longer$indicators=="Livestock presence", "ns_GLLVM", as.character(even_longer$variable))
# Bio
even_longer$color_var <- ifelse(even_longer$variable=="Bio" &
                    (even_longer$indicators=="Biomass yield" | even_longer$indicators== "Digestibility" |
                     even_longer$indicators== "Root mass" | even_longer$indicators== "Plant richness" |
                     even_longer$indicators== "less Plant path." | even_longer$indicators== "less Herbivory" |
                     even_longer$indicators== "N2 fixation" |
```

```
                    even_longer$indicators== "Earthworms" | even_longer$indicators== "less
Topsoil P" |
                    even_longer$indicators== "Edible Plants" | even_longer$indicators== "Iconic
fungi" |
                    even_longer$indicators== "Livestock presence" | even_longer$indicators==
"Aesthetic" |
                    even_longer$indicators== "less N2O emissions" | even_longer$indicators== "less
Weeds"),
                 "ns_GLLVM", as.character(even_longer$color_var))
# We
even_longer$color_var <- ifelse(even_longer$variable=="We" &
                  (  even_longer$indicators== "Root mass" |
                     even_longer$indicators== "less Plant path." | even_longer$indicators== "less
Herbivory" |
                     even_longer$indicators== "AM fungi" |
                     even_longer$indicators== "less Topsoil P" |
                     even_longer$indicators== "Iconic fungi"),
                 "ns_GLLVM", as.character(even_longer$color_var))
str(even_longer$color_var)
even_longer$color_var <- as.character(even_longer$color_var)


#### for empty symbols if SEM path insignificant:


el_2 <-even_longer[order(even_longer$indicators),]
rownames(el_2) <- 1:204
el_2$color_var3 <- c("Ext", "ns","ns","ns",     "ns","ns","ns","ns",      "We", "ns","ns","ns", #Aesthetic
             "ns", "Ext", "ns","ns",    "ns", "Bio", "ns","ns",    "ns","We", "ns","ns", #AM fungi
             "Ext", "Ext", "ns","ns",    "ns", "Bio", "ns","ns",    "ns","We", "ns","ns", #Biomass yield
             "Ext", "ns","Ext","ns",     "ns","ns","ns","ns",      "We", "ns","We","ns", #Digestibility
             "Ext", "ns","ns","ns",      "ns","ns","ns","ns",      "ns", "ns","ns","ns", #Earthworms
             "ns", "ns","Ext","ns",      "ns","ns","ns","ns",      "ns", "ns","We","ns", #Edible Plants
             "ns", "Ext", "ns","ns",     "ns", "Bio", "ns","ns",    "ns","We", "ns","ns", #Iconic fungi
             "Ext", "ns","ns","ns",      "ns","ns","ns","ns",      "ns", "ns","ns","ns", #less Herbivory
```

```
"ns", "Ext", "Ext", "Ext",   "ns", "Bio", "ns", "ns",   "ns", "We", "We", "We", #less N leach
"Ext", "Ext", "Ext", "Ext",  "ns", "Bio", "ns", "ns",   "We", "We", "We", "We", #less N2O
"Ext", "ns","ns","ns",       "ns","ns","ns","ns",       "ns", "ns","ns","ns", #less Plant path
"ns", "Ext", "ns","Ext",     "ns", "Bio", "ns","ns",    "ns","We", "ns","We", #less Surface P
"ns", "ns","ns","ns",        "ns","ns","ns","ns",       "We", "ns","ns","ns", #less Weeds
"ns", "Ext","Ext","ns",      "ns","Bio","ns","ns",      "ns", "We","We","ns", #Livestock presence
"ns", "ns","ns","Ext",       "ns","ns","ns","ns",       "We", "ns","ns","We", #N2 fixation
"ns", "Ext","Ext","Ext",     "ns","Bio","ns","ns",      "ns", "We","We","We", #Plant richness
"ns", "ns","Ext","ns",       "ns","ns","ns","ns",       "ns", "ns","We","ns")#Root mass

el_2$color_var3 <- as.factor(el_2$color_var3)

el_2$shapes <- ifelse(el_2$variable == "Ext"& el_2$color_var3 == "Ext", 21, ifelse
               (el_2$variable=="Bio"& el_2$color_var3 == "Bio", 22, ifelse
                (el_2$variable =="We"& el_2$color_var3 == "We", 24, ifelse(
                 ((el_2$variable =="Ext") & (el_2$color_var3 == "ns")), 1, ifelse(
                  (el_2$variable =="Bio" & el_2$color_var3 == "ns"), 0, 2)))))

el_2$shapes <- as.factor(el_2$shapes)

el_2 <- el_2[order(el_2$variable, decreasing=T),]

############ plotting it

library(ggplot2)
library(dplyr)
library(forcats)
library(GGally)
```

```
el_2 %>%
  mutate(categ = fct_relevel(categ,
                  "via grazing intensity","via Nr. of cuts", "via fertilizer N", "Direct"),
        indicators = fct_relevel(indicators,
                  "Biomass yield", "Digestibility", "Root mass", "Plant richness",
                  "less Plant path.", "less Herbivory", "less Weeds", "N2 fixation",
                  "AM fungi", "Earthworms", "less N leaching", "less Surface P",
                  "less N2O emissions", "Edible Plants", "Iconic fungi", "Livestock presence",
"Aesthetic"),
        variable=fct_relevel(variable, "Ext", "We", "Bio")) %>%
  ggplot(aes(x=value, y=categ, shape=shapes, fill=color_var3))+
  theme_test(base_size=14)+
  geom_stripped_rows()+
  geom_vline(xintercept = 0, color="grey")+
  geom_jitter(alpha=0.6, size=2.5, width=0, height=0.0)+
  scale_shape_manual(values=c(0, 1, 2, 21, 22, 24))+
  scale_fill_manual(values=c( "#F3B403", "#DE5518", NA, "cadetblue4"),
            labels=c("Production System: Organic", "Eco-Scheme: Yes", "Harvest Type: Pasture", ""))+
  ylab("")+
  xlab("standardized effects from SEM")+
  facet_wrap(~indicators, ncol=6)
```

R-code for calculating the confidence intervals to the mean log response ratio

#Calculation of Confidence Intervals (CI) to the mean log response ratio (MLRR)

#Note: the code is given for the CI to the MLRR regarding Production system, Eco-scheme, and Harvest type, each time split into Provisioning ES, Supporting/Regulating ES, and Cultural ES. The overall MLRR simply omits the grouping into Provisioning, Supporting/Regulating, and Cultural.

#Confidence intervals are calculated following Davison and Hinkley (1997), p. 194

```
#-------------------------------------------------------------------------------------------#

#-------------------------------------------------------------------------------------------#

#Data

d.data1 <- read.table("MiceFIlledForLRR_Fig5.txt",header=TRUE,sep=";")

d.data <- d.data1[, c(1:25)]

str(d.data)   #86 obs


#-------------------------------------------------------------------------------------------#

#-------------------------------------------------------------------------------------------#

#-------------------------------------------------------------------------------------------#

#Basic calculations


#-------------------------------------------------------------------------------------------#

#Means per 8 management groups


d.means <- aggregate(x = d.data[, 1:22], by = list(d.data[, 25], d.data[, 24], d.data[, 23] ), FUN = "mean")

colnames(d.means)[1:3] <- c("Harvest_type", "Eco_scheme", "Prod_system")


#-------------------------------------------------------------------------------------------#

#Vector of reversed functions (where minimal values are regarded as of positive benefit)

less.vect <- c(1,1,1,1,1,-1,-1,-1,1,-1,1,1,1,-1,-1,-1,1,1,-1,1,1,1)

names(less.vect) <- colnames(d.means)[4:25]
```

```
#-----------------------------------------------------------------------------------------------#

#Function to group LRRs into CICES and calculation of MLRR

MLLR.func <- function(LRRS)

{

  #Provisioning

  d.1.1.3.1 <- mean(LRRS[c("Biomass","Digestibility")])


  MLRR.1 <- d.1.1.3.1


  #Supporting/Regulating

  d.2.2.1.1 <- as.numeric(LRRS["Rootmass"])

  d.2.2.1.3 <- as.numeric(LRRS["Soil_compact"])

  d.2.2.2.1 <- as.numeric(LRRS["Nectar"])

  d.2.2.2.3 <- as.numeric(LRRS["Plant_richness"])

  d.2.2.3.1 <- mean(LRRS[c("Plant_path","Herbivory","Weeds")])

  d.2.2.4.1 <- mean(LRRS[c("N_fix","AM_Fungi","MBC","Heavy_metals","Earthworms")])

  d.2.2.5.1 <- mean(LRRS[c("N_leach","P")])

  d.2.2.6.1 <- mean(LRRS[c("N2O","C_stock")])


  MLRR.2 <- mean(c(d.2.2.1.1,d.2.2.1.3,d.2.2.2.1,d.2.2.2.3,d.2.2.3.1,d.2.2.4.1,d.2.2.5.1,d.2.2.6.1))


  #Cultural

  d.3.1.1.1 <- mean(LRRS[c("Edible_plants","Iconic_fungi")])

  d.3.1.2.3 <- as.numeric(LRRS["Cow_days"])

  d.3.1.2.4 <- as.numeric(LRRS["Aesthetics"])


  MLRR.3 <- mean(c(d.3.1.1.1,d.3.1.2.3,d.3.1.2.4))


  MLRR.4 <-
mean(c(d.1.1.3.1,d.2.2.1.1,d.2.2.1.3,d.2.2.2.1,d.2.2.2.3,d.2.2.3.1,d.2.2.4.1,d.2.2.5.1,d.2.2.6.1,d.3.1.1.1
,d.3.1.2.3,d.3.1.2.4))
```

```
 MLRR.out <- c(MLRR.1,MLRR.2,MLRR.3, MLRR.4)
 names(MLRR.out) <- c("Provisioning","Prov./Supp.","Cultural", "Overall")
 MLRR.out
}
#---------------------------------------------------------------------------------------------#

#Split data to 8 management groups for re-sampling
d.1 <- subset(d.data, Prod_system=="non-org" & Eco_scheme=="no"  & Harvest_type=="meadow")
d.2 <- subset(d.data, Prod_system=="non-org" & Eco_scheme=="no"  & Harvest_type=="pasture")
d.3 <- subset(d.data, Prod_system=="non-org" & Eco_scheme=="yes" & Harvest_type=="meadow")
d.4 <- subset(d.data, Prod_system=="non-org" & Eco_scheme=="yes" & Harvest_type=="pasture")
d.5 <- subset(d.data, Prod_system=="organic" & Eco_scheme=="no"  & Harvest_type=="meadow")
d.6 <- subset(d.data, Prod_system=="organic" & Eco_scheme=="no"  & Harvest_type=="pasture")
d.7 <- subset(d.data, Prod_system=="organic" & Eco_scheme=="yes" & Harvest_type=="meadow")
d.8 <- subset(d.data, Prod_system=="organic" & Eco_scheme=="yes" & Harvest_type=="pasture")

#---------------------------------------------------------------------------------------------#
#---------------------------------------------------------------------------------------------#
#---------------------------------------------------------------------------------------------#
#MLRR and CI to Production system: organic versus non-organic
LRR <- colMeans( log(d.means[5:8,4:25]/d.means[1:4,4:25]) ) * less.vect

MLRR.orig <- MLLR.func(LRRS=LRR) ; MLRR.orig

#---------------------------------------------------------------------------------------------#
#Bootstrap CI
#Initialise Matrix for re-sampling
nr <- 1000
d.boot <- matrix(NA, nrow=nr, ncol=4)
#-----------------------------------------------------#
#Sample loop
for (i in 1:nr)
```

```
{
  #Sample
  s.1 <- d.1[sample(1:nrow(d.1), size=nrow(d.1), replace=TRUE),]
  s.2 <- d.2[sample(1:nrow(d.2), size=nrow(d.2), replace=TRUE),]
  s.3 <- d.3[sample(1:nrow(d.3), size=nrow(d.3), replace=TRUE),]
  s.4 <- d.4[sample(1:nrow(d.4), size=nrow(d.4), replace=TRUE),]
  s.5 <- d.5[sample(1:nrow(d.5), size=nrow(d.5), replace=TRUE),]
  s.6 <- d.6[sample(1:nrow(d.6), size=nrow(d.6), replace=TRUE),]
  s.7 <- d.7[sample(1:nrow(d.7), size=nrow(d.7), replace=TRUE),]
  s.8 <- d.8[sample(1:nrow(d.8), size=nrow(d.8), replace=TRUE),]

  d.sample <- rbind(s.1,s.2,s.3,s.4,s.5,s.6,s.7,s.8)

  #Means per 8 management groups

  d.means <- aggregate(x = d.sample[, 1:22], by = list(d.sample[, 25], d.sample[, 24], d.sample[, 23] ),
FUN = "mean")
  colnames(d.sample)[1:3] <- c("Harvest_type", "Eco_scheme", "Prod_system")

  #----------------------------------------------------#
  #LRRs
  LRR <- colMeans( log(d.means[5:8,4:25]/d.means[1:4,4:25]) ) * less.vect

  #Group into CICES and calculation of MLRR
  d.boot[i,] <- MLLR.func(LRRS=LRR)
}
#----------------------------------------------------#
#CIs
lower <- function(data) {sort(data, decreasing=FALSE)[0.025*length(na.omit(data))]}
upper <- function(data) {sort(data, decreasing=FALSE)[(1-0.025)*length(na.omit(data))]}

lower.lim <- apply(d.boot, MARGIN=2, FUN=lower)
```

```
upper.lim <- apply(d.boot, MARGIN=2, FUN=upper)


#Basic CI (also termed Pivotal CI)

2*MLRR.orig - upper.lim

2*MLRR.orig - lower.lim


#---------------------------------------------------------------------------------------------#

#---------------------------------------------------------------------------------------------#

#---------------------------------------------------------------------------------------------#

#MLRR and CI to Eco-scheme: yes versus no


d.means <- aggregate(x = d.data[, 1:22], by = list(d.data[, 25], d.data[, 24], d.data[, 23] ), FUN =
"mean")

colnames(d.means)[1:3] <- c("Harvest_type", "Eco_scheme", "Prod_system")




LRR.A <- log(d.means[3:4,4:25]/d.means[1:2,4:25])

LRR.B <- log(d.means[7:8,4:25]/d.means[5:6,4:25])


LRR <- colMeans( rbind(LRR.A,LRR.B) ) * less.vect


MLRR.orig <- MLLR.func(LRRS=LRR) ; MLRR.orig


#---------------------------------------------------------------------------------------------#

#Bootstrap CI

#Initialise Matrix for re-sampling

nr <- 1000

d.boot <- matrix(NA, nrow=nr, ncol=4)


#----------------------------------------------------#

#Sample loop

for (i in 1:nr)
```

```
{
  #Sample
  s.1 <- d.1[sample(1:nrow(d.1), size=nrow(d.1), replace=TRUE),]
  s.2 <- d.2[sample(1:nrow(d.2), size=nrow(d.2), replace=TRUE),]
  s.3 <- d.3[sample(1:nrow(d.3), size=nrow(d.3), replace=TRUE),]
  s.4 <- d.4[sample(1:nrow(d.4), size=nrow(d.4), replace=TRUE),]
  s.5 <- d.5[sample(1:nrow(d.5), size=nrow(d.5), replace=TRUE),]
  s.6 <- d.6[sample(1:nrow(d.6), size=nrow(d.6), replace=TRUE),]
  s.7 <- d.7[sample(1:nrow(d.7), size=nrow(d.7), replace=TRUE),]
  s.8 <- d.8[sample(1:nrow(d.8), size=nrow(d.8), replace=TRUE),]

  d.sample <- rbind(s.1,s.2,s.3,s.4,s.5,s.6,s.7,s.8)

  #Means per 8 management group
  d.means <- aggregate(x = d.sample[, 1:22], by = list(d.sample[, 25], d.sample[, 24], d.sample[, 23] ),
FUN = "mean")
  colnames(d.sample)[1:3] <- c("Harvest_type", "Eco_scheme", "Prod_system")

  #-----------------------------------------------------#
  #LRRs
  LRR.A <- log(d.means[3:4,4:25]/d.means[1:2,4:25])
  LRR.B <- log(d.means[7:8,4:25]/d.means[5:6,4:25])

  LRR <- colMeans( rbind(LRR.A,LRR.B) ) * less.vect

  #Group into CICES and calculation of MLRR
  d.boot[i,] <- MLLR.func(LRRS=LRR)
}
#-----------------------------------------------------#
#CIs
lower <- function(data) {sort(data, decreasing=FALSE)[0.025*length(na.omit(data))]}
upper <- function(data) {sort(data, decreasing=FALSE)[(1-0.025)*length(na.omit(data))]}
```

```
lower.lim <- apply(d.boot, MARGIN=2, FUN=lower)
upper.lim <- apply(d.boot, MARGIN=2, FUN=upper)

#Basic CI (also termed Pivotal CI)
2*MLRR.orig - upper.lim
2*MLRR.orig - lower.lim

#----------------------------------------------------------------------------------------------#
#----------------------------------------------------------------------------------------------#
#----------------------------------------------------------------------------------------------#
#MLRR and CI to Harvest type: pasture versus meadow

d.means <- aggregate(x = d.data[, 1:22], by = list(d.data[, 25], d.data[, 24], d.data[, 23] ), FUN =
"mean")
colnames(d.means)[1:3] <- c("Harvest_type", "Eco_scheme", "Prod_system")


LRR.A <- log(d.means[2,4:25]/d.means[1,4:25])
LRR.B <- log(d.means[4,4:25]/d.means[3,4:25])
LRR.C <- log(d.means[6,4:25]/d.means[5,4:25])
LRR.D <- log(d.means[8,4:25]/d.means[7,4:25])

LRR <- colMeans( rbind(LRR.A,LRR.B,LRR.C,LRR.D) ) * less.vect

MLRR.orig <- MLLR.func(LRRS=LRR) ; MLRR.orig

#----------------------------------------------------------------------------------------------#
#Bootstrap CI
#Initialise Matrix for re-sampling
nr <- 1000
d.boot <- matrix(NA, nrow=nr, ncol=4)
```

```
#----------------------------------------------------#
#Sample loop
for (i in 1:nr)
{
  #Sample
  s.1 <- d.1[sample(1:nrow(d.1), size=nrow(d.1), replace=TRUE),]
  s.2 <- d.2[sample(1:nrow(d.2), size=nrow(d.2), replace=TRUE),]
  s.3 <- d.3[sample(1:nrow(d.3), size=nrow(d.3), replace=TRUE),]
  s.4 <- d.4[sample(1:nrow(d.4), size=nrow(d.4), replace=TRUE),]
  s.5 <- d.5[sample(1:nrow(d.5), size=nrow(d.5), replace=TRUE),]
  s.6 <- d.6[sample(1:nrow(d.6), size=nrow(d.6), replace=TRUE),]
  s.7 <- d.7[sample(1:nrow(d.7), size=nrow(d.7), replace=TRUE),]
  s.8 <- d.8[sample(1:nrow(d.8), size=nrow(d.8), replace=TRUE),]

  d.sample <- rbind(s.1,s.2,s.3,s.4,s.5,s.6,s.7,s.8)

  #Means per 8 management groups
  d.means <- aggregate(x = d.sample[, 1:22], by = list(d.sample[, 25], d.sample[, 24], d.sample[, 23] ),
FUN = "mean")
  colnames(d.sample)[1:3] <- c("Harvest_type", "Eco_scheme", "Prod_system")

  #----------------------------------------------------#
  #LRRs
  LRR.A <- log(d.means[2,4:25]/d.means[1,4:25])
  LRR.B <- log(d.means[4,4:25]/d.means[3,4:25])
  LRR.C <- log(d.means[6,4:25]/d.means[5,4:25])
  LRR.D <- log(d.means[8,4:25]/d.means[7,4:25])

  LRR <- colMeans( rbind(LRR.A,LRR.B,LRR.C,LRR.D) ) * less.vect

  #Group into CICES and calculation of MLRR
  d.boot[i,] <- MLLR.func(LRRS=LRR)
```

```
}
#-----------------------------------------------------#
#CIs
lower <- function(data) {sort(data, decreasing=FALSE)[0.025*length(na.omit(data))]}
upper <- function(data) {sort(data, decreasing=FALSE)[(1-0.025)*length(na.omit(data))]}

lower.lim <- apply(d.boot, MARGIN=2, FUN=lower)
upper.lim <- apply(d.boot, MARGIN=2, FUN=upper)

#Basic CI (also termed Pivotal CI)
2*MLRR.orig - upper.lim
2*MLRR.orig - lower.lim
```

Supplementary Note. Questionnaire.

Management Fragebogen:

**Name: ……**	**Plot Nummer *ServiceGrass*: …….**

Datum: ……	Name Befragende: ……

**1.**	**Nutzungen**			*falls Weide* – **Tiere:**			
	**Nutzungen** in 2020 (Silage, Heu, Weide, etc..)	**Datum + Anzahl Tage**	**Stunden /Tag**	**Anzahl**	**Art**	**Alter**	**Zufütterung** (Ja/Nein)
	1.						
	2.						
	3.						
	4.						
	5.						
	6.						
	7.						
	8.						

**2.**	**Pflege**
	Wurde eine der Aktivitäten unten durchgeführt?<br>☐ **Mechanisches Entfernen** von Gehölzen oder Unkraut (stechen, ziehen)<br>☐ **Pflanzenschutzmittel** und/oder **Rodentizid**<br>☐ Andere Massnahmen: ……

**3.**	**Dünger**				
	**Art der Düngung**<br>***Gülle*** (gemischt oder haupts. Urin), ***Mist*** (konzentriert, strohreich, Hof-), ***Mineraldünger***, ***Klärschlamm***, ***Jauche***, Anderes	NPK Dünger **Zusammensetzung** (falls benutzt) oder Wenn bekannt, Kg N (/Fläche)	**Menge** (m^3, kg, Tonnen). ***Für die Parzelle oder pro ha oder m^2 (EINHEIT dazu!)***	**Verdün-nung** der Gülle?	**Datum** (2020)
	1.				
	2.				

	3.				
	4.				
	5.				
	6.				

Management Questionnaire

Plot Nubmer *ServiceGrass*: …….

Date: ……	Interviewer: ……

**1.**	**Use of grassland**		*In case of pasture* – **Animals:**				
	**Uses** in 2020 (Silage, Hay, Pasture, etc..)	**Date + Nr. Days**	**Hours/day**	**Number**	**Species**	**Age**	**Additional feeding** (Y/N)
	1.						
	2.						
	3.						
	4.						
	5.						
	6.						
	7.						
	8.						

2. Other management

Was any of the following activities done?

☐ **Mechanical** disposal of weeds or woody species

☐ **Pesticide application**

☐ Other: ……

3. Fertilization

**Kind of fertilization** *slurry* (mixed or mainly urine), *manure* (concentrated, straw-rich), other	NPK fertilizer **Contents** (in case applied) or if known, Kg N (/area)	**Amount** (m^3, kg, tons). ***Per parcel or ha or m^2 (ADD)***	**Dilution** of slurry?	**Date** (2020)
1.				

	2.				
	3.				
	4.				
	5.				
	6.				

Supplementary Note. Informed Consent.

The following pages show the declaration of consent signed by the participating farmers, in German as well as translated to English.

Einverständniserklärung zur Erhebung, Verknüpfung und Bearbeitung von Daten

Detailinformationen

Die vorliegende Datenerhebung, die aus Felderhebungen und einer betrieblichen Befragung besteht, wird zu Forschungszwecken von der Eidgenössischen Technischen Hochschule Zürich (ETH) und von der Forschungsanstalt Agroscope im Rahmen der Forschungsprojekte *ServiceGrass* und *IndiGras* durchgeführt. Beide Forschungsprojekte dienen der **Evaluation der privaten und öffentlichen Ökosystemleistungen und der Multifunktionalität von Schweizer Grasland.** Das Projekt *IndiGras* ist eine Ergänzung zum bestehenden Projekt *ServiceGrass* und wird die bereits erhobenen Daten auswerten, um ein Indikatorsystem zur Bewertung der Ökosystemleistungen von Landwirtschaftsbetrieben mit einer abgestuften Bewirtschaftungsintensität ihrer Graslandflächen abzuleiten. Eine erneute Datenerhebung ist im Rahmen von *IndiGras* nicht geplant.

Die im Rahmen der Feldarbeiten und Befragung erhobenen Daten[3] werden von der ETH und Agroscope **ohne Namen und Adresse**, sondern nur mit einer Betriebsnummer (pseudonymisiert) aufbewahrt und bearbeitet. Die Ergebnisse der Forschungsarbeiten werden in wissenschaftlichen und praxisorientierten Publikationen veröffentlicht. Die Veröffentlichung erfolgt in einer Weise, dass Personen und Betriebe nicht bestimmbar und Rückschlüsse auf das Verhalten einzelner Betriebe nicht möglich sind.

Die Teilnahme der Betriebsleitenden an den im Rahmen dieser Forschungsprojekte durchgeführten Datenerhebungen (Felderhebungen und Befragung) ist freiwillig. Eine Verwendung der erhobenen Daten für administrative Kontrollzwecke ist ausgeschlossen**.**
Die erhobenen Daten werden mit Strukturdaten (Allgemeine Betriebsangaben, Flächen, Tierbestände, Arbeitskräfte und Direktzahlungen[4]) aus dem **agrarpolitischen Informationssystem AGIS** des Bundesamtes für Landwirtschaft (BLW) sowie mit Geoinformationsdaten zu Grösse und Lage der Parzellen, bereitgestellt vom Amt für Landwirtschaft des Kantons Solothurn, **verknüpft**. Diese Verknüpfung verhindert die doppelte Erhebung der gleichen Daten und reduziert damit den Aufwand für die Studienteilnehmenden.

Die einzelbetrieblichen Daten der Felderhebung und der Befragungen sollen spätestens nach Beendigung beider Forschungsprojekte anonymisiert veröffentlicht werden, um eine zukünftige Nutzung für Forschungszwecke zu ermöglichen.

Ihre Daten werden in Übereinstimmung mit dem Schweizer Bundesgesetz über den Datenschutz bearbeitet und aufbewahrt.

Die nachfolgende Einverständniserklärung stellt die rechtmässige Grundlage für die Verarbeitung Ihrer personenbezogenen Daten in Übereinstimmung mit dem schweizerischen Datenschutzgesetz dar.

Sie können sich jederzeit **an den Projektleitenden wenden, um Ihre Einwilligung zu widerrufen** nachdem sie die Einverständniserklärung unterzeichnet haben (ohne etwaige Nachteile für Sie)**.**

Dr. Valentin Klaus
Professur für Graslandwissenschaften
Universitätstrasse 2, 8092 Zürich
Telefon: 044 632 49 32; E-Mail: valentin.klaus@usys.ethz.ch

[3] Biophysikalische Daten, Daten zur Graslandbewirtschaftung, Kennzahlen zum Betrieb (vorwiegend produktionstechnische Daten), Soziodemografische Daten

[4] Die Direktzahlungsdaten werden nur von Agroscope verwendet. Die ETH Zürich hat keinen Zugriff auf diese Daten.

Einverständniserklärung

Mit der Teilnahme an den Felderhebungen und der vorliegenden Umfrage erkläre ich mich damit einverstanden, dass

meine im Rahmen der Felderhebungen und der Befragung bekannt gegebenen Daten:

- mit Strukturdaten (Allgemeine Betriebsangaben, Flächen, Tierbestände, Arbeitskräfte und Direktzahlungen) aus dem AGIS (agrarpolitisches Informationssystem des BLW) **sowie mit Geoinformationsdaten zu Grösse und Lage der Parzellen verknüpft** werden;
- verknüpft mit Strukturdaten (Allgemeine Betriebsangaben, Flächen, Tierbestände, Arbeitskräfte und Direktzahlungen[5]) aus dem AGIS sowie mit Geoinformationsdaten zu Grösse und Lage der Parzellen, für **Forschungszwecke von der ETH und Agroscope im Rahmen der Forschungsprojekte *ServiceGrass* und *IndiGrass* verwendet** werden;
- von der ETH und Agroscope ohne Namen und Adresse (pseudonymisiert) bearbeitet werden, in Ergebnisse und Auswertungen einfliessen, die in einer Weise veröffentlicht werden, dass **Personen und Betriebe nicht bestimmbar** und Rückschlüsse auf das Verhalten einzelner Betriebe nicht möglich sind.
- (exklusive der AGIS-Daten) spätestens nach Beendigung beider Forschungsprojekte **anonymisiert veröffentlicht** werden, um eine zukünftige Nutzung für Forschungszwecke zu ermöglichen.

☐ **Ja, ich erkläre mich damit einverstanden.**

☐ **Nein, ich stimme nicht zu und kann an der Datenerhebung somit nicht teilnehmen.**

Datum, Ort: ....

Vor- und Nachname: ....

Unterschrift:

---

[5] Die Direktzahlungsdaten werden nur von Agroscope verwendet. Die ETH Zürich hat keinen Zugriff auf diese Daten.

Declaration of consent to the collection, linking and processing of data

Detailed information

The present data collection, which consists of field surveys and a company survey, is carried out for research purposes by the Swiss Federal Institute of Technology Zurich (ETH) and by the Agroscope research institute as part of the *ServiceGrass* and *IndiGras* research projects. Both research projects serve to evaluate the **private and public ecosystem services and the multifunctionality of Swiss grasslands**. The *IndiGras* project is an addition to the existing ServiceGrass project and will evaluate the data already collected in order to derive an indicator system for assessing the ecosystem services of farms with a graded management intensity of their grassland areas. There are no plans to collect data again as part of *IndiGras*.

The data[3] collected as part of the field work and survey is stored and processed by ETH and Agroscope **without a name and address**, but only with a company number (pseudonymized). The results of the research work are published in scientific and practice-oriented publications. The publication is carried out in such a way that people and companies cannot be identified and conclusions about the behavior of individual companies are not possible.

The participation of management in the data collection (field surveys and surveys) carried out as part of these research projects is voluntary. Use of the data collected for administrative control purposes is excluded.

The **data[3] collected is linked to structural data** (general farm information, areas, livestock, workforce and direct payments[4]) **from the agricultural policy information system AGIS** of the Federal Office of Agriculture (BLW) as well as to geoinformation data on the size and location of the plots, provided by the Office of Agriculture of the Canton of Solothurn . This link prevents the same data from being collected twice and thus reduces the effort for study participants.

The individual company data from the field survey and the surveys should be published anonymously at the latest after both research projects have been completed in order to enable future use for research purposes.

Your data will be processed and stored in accordance with the Swiss Federal Data Protection Act.

The following declaration of consent represents the lawful basis for the processing of your personal data in accordance with the Swiss Data Protection Act.

You can **contact the project leader at any time to withdraw your consent** after signing the consent form (without any disadvantage to you).

Dr. Valentin Klaus
Professur für Graslandwissenschaften
Universitätstrasse 2, 8092 Zürich
Telefon: 044 632 49 32; E-Mail: valentin.klaus@usys.ethz.ch

[3] Biophysical data, grassland management data, operational key figures (mainly production data), socio-demographic data
[4] The direct payment data is only used by Agroscope. ETH Zurich has no access to this data.

Declaration of consent

By participating in the field surveys and this survey, I agree that

my data provided during the field surveys and the survey:

- Will be connected with structural data (general farm information, areas, livestock, workforce and direct payments) from the AGIS (agropolitical information system of the BLW[3]) **as well as with geodata concerning size and location of the parcels;**
- Will be **used for scientific purposes from ETH and Agroscope within the scientific projects SericeGrass and IndiGras**, connected with structural data (general farm information, areas, livestock, workforce and direct payments[3]) from the AGIS (agropolitical information system of the BLW) as well as with geodata concerning size and location of the parcels;
- Will be processed by ETH and Agroscope without name and adress (pseudonymized), will be integrated into Results that will be published in such a way, **that persons and farms can not be identified** and no conclusions on the behavior of single farms are possible.
- Will be **published** (excluding the AGIS-data) at the latest after both the projects are closed, **in an anonymized way**, to make future scientific uses possible.

☐ **Yes, I agree.**

☐ **No, I do not agree and can thus not participate in the data collection.**

Date, Place: ....

Name and Family Name: ....

Signature:

---

[3] The direct payment data is only used by Agroscope. ETH Zurich has no access to this data.
