# Supplementary material for: Effects of management practices on the ecosystem-service multifunctionality of temperate grasslands
Source: Nat Commun. 2024 May 7;15:3829. doi: 10.1038/s41467-024-48049-y (PMC11076620; doi:10.1038/s41467-024-48049-y)
Supplement: Supplementary file 2 — Reporting Summary [file 41467_2024_48049_MOESM2_ESM.pdf]

nature portfolio

Corresponding author(s): Franziska Richter

Last updated by author(s): Mar 8, 2024

Reporting Summary

Nature Portfolio wishes to improve the reproducibility of the work that we publish. This form provides structure for consistency and transparency in reporting. For further information on Nature Portfolio policies, see our Editorial Policies and the Editorial Policy Checklist.

Statistics

For all statistical analyses, confirm that the following items are present in the figure legend, table legend, main text, or Methods section.

n/a	Confirmed	
☐	☒	The exact sample size (*n*) for each experimental group/condition, given as a discrete number and unit of measurement
☐	☒	A statement on whether measurements were taken from distinct samples or whether the same sample was measured repeatedly
☐	☒	The statistical test(s) used AND whether they are one- or two-sided<br>*Only common tests should be described solely by name; describe more complex techniques in the Methods section.*
☐	☒	A description of all covariates tested
☐	☒	A description of any assumptions or corrections, such as tests of normality and adjustment for multiple comparisons
☐	☒	A full description of the statistical parameters including central tendency (e.g. means) or other basic estimates (e.g. regression coefficient) AND variation (e.g. standard deviation) or associated estimates of uncertainty (e.g. confidence intervals)
☐	☒	For null hypothesis testing, the test statistic (e.g. *F*, *t*, *r*) with confidence intervals, effect sizes, degrees of freedom and *P* value noted<br>*Give P values as exact values whenever suitable.*
☐	☒	For Bayesian analysis, information on the choice of priors and Markov chain Monte Carlo settings
☐	☒	For hierarchical and complex designs, identification of the appropriate level for tests and full reporting of outcomes
☐	☒	Estimates of effect sizes (e.g. Cohen's *d*, Pearson's *r*), indicating how they were calculated

Our web collection on statistics for biologists contains articles on many of the points above.

Software and code

Policy information about availability of computer code

Data collection	No software was used to collect data
Data analysis	For data analysis, the software R version 4.2.0. R packages used: gllvm version 1.4.2; ggplot2 3.4.0 for figures; mice v. 3.15.0 for gap-filling; FUNGuildR version 0.2.0. For Microbial analyses: VSEARCH v.2.21.1

For manuscripts utilizing custom algorithms or software that are central to the research but not yet described in published literature, software must be made available to editors and reviewers. We strongly encourage code deposition in a community repository (e.g. GitHub). See the Nature Portfolio guidelines for submitting code & software for further information.

Data

Policy information about availability of data

All manuscripts must include a data availability statement. This statement should provide the following information, where applicable:

- Accession codes, unique identifiers, or web links for publicly available datasets
- A description of any restrictions on data availability
- For clinical datasets or third party data, please ensure that the statement adheres to our policy

The ecosystem service-indicator, environmental, and management data generated in this study have been deposited in the ETH research collection under accession code ethz-b-000663689 [https://www.research-collection.ethz.ch/handle/20.500.11850/663689]. The sequencing data is deposited in the European Nucleotide Archive under the accession number PRJEB72428 [https://www.ebi.ac.uk/ena/browser/view/PRJEB72428]. Further Databases used in this study: Unite v.83 database (Kõljalg U, Nilsson HR, Schigel D, Tedersoo L, Larsson K-H, May TW, Taylor AFS, Jeppesen TS, Frøslev TG, Lindahl BD, Põldmaa K, Saar I, Suija A, Savchenko

A, Yatsiuk I, Adojaan K, Ivanov F, Piirmann T, Pöhönen R, Zirk A, Abarenkov K. The Taxon Hypothesis Paradigm—On the Unambiguous Detection and Communication of Taxa. Microorganisms. 2020; 8(12):1910. DOI: 10.3390/microorganisms8121910), FUNGuild database (Nguyen NH, Song Z, Bates ST, et al (2016) FUNGuild: An open annotation tool for parsing fungal 648 community datasets by ecological guild. Fungal Ecol 20:241–248. 649 https://doi.org/10.1016/j.funeco.2015.06.006), Data on Nectar availability from Baude, M., Kunin, W., Boatman, N. et al. Historical nectar assessment reveals the fall and rise of floral resources in Britain. Nature 530, 85–88 (2016). https://doi.org/10.1038/nature16532 and Filipiak, M., Walczyńska, A., Denisow, B., Petanidou, T. & Ziółkowska, E. Phenology and production of pollen, nectar, and sugar in 1612 plant species from various environments. Ecology 103, 2021–2022 (2022), DOI for data: 10.5281/zenodo.5862277. The digital elevation model used is from the European Union (2018): Copernicus Land Monitoring Service, European Environment Agency (EEA) [WWW Document], (accessed 12.25.20).

Research involving human participants, their data, or biological material

Policy information about studies with human participants or human data. See also policy information about sex, gender (identity/presentation), and sexual orientation and race, ethnicity and racism.

Reporting on sex and gender	No information on sex or gender of any humans was collected.
Reporting on race, ethnicity, or other socially relevant groupings	We did not use any variables categorizing humans
Population characteristics	No data was collected about humans
Recruitment	We did not conduct research about humans
Ethics oversight	ETH Zürich

Note that full information on the approval of the study protocol must also be provided in the manuscript.

Field-specific reporting

Please select the one below that is the best fit for your research. If you are not sure, read the appropriate sections before making your selection.

☐ Life sciences ☐ Behavioural & social sciences ☒ Ecological, evolutionary & environmental sciences

For a reference copy of the document with all sections, see nature.com/documents/nr-reporting-summary-flat.pdf

Ecological, evolutionary & environmental sciences study design

All studies must disclose on these points even when the disclosure is negative.

Study description	86 permanent grasslands managed by farmers were investigated in the Canton of Solothurn, Switzerland. Factorial design, in which grasslands could be either grazed or mown (Harvest type), organic or non-organic (Production System) or managed extensively or intensively (Eco-scheme extensive). All eight combinations of the three management aspects where included.
Research sample	Several samples were collected - soil, root biomass, aboveground biomass, legume samples. Additionally, many other analyses were carried out on site such as vegetation survey, counting earthworms, herbivory assessment. Depending on the measurement, different replicate sizes were chosen. 20 soil samples were taken per grassland, but pooled to attain a composite sample. two vegetation assessments were carried out, earthworms were counted in three locations, and the mean value used for further calculations. The rationale for the sample choice was the following: We were aiming to measure as many ecosystem services with on-site material as possible. Thus, we collected soil samples to assess nutrient status, aboveground biomass to assess forage quantity and quality, and we sampled the vegetation as an indicator for the ecosystem service "habitat for nursery population", counted earthworms as a proxy for soil structure, herbivory was assessed as an indicator for "pest resistance". The population the samples are supposed to represent are the 86 grasslands (one mean value of replicates within a plot as final information per grassland). To gain information on the nectar content of plant species per cover, we used datasets collected in studies by Baude, M. et al.: Historical nectar assessment reveals the fall and rise of floral resources in Britain. Nature 530, 85–88 (2016), Supplementary Table 13 and Filipiak, M., Walczyńska, A., Denisow, B., Petanidou, T. & Ziółkowska, E. Phenology and production of pollen, nectar, and sugar in 1612 plant species from various environments. Ecology 103, 2021–2022 (2022), DOI for data: 10.5281/zenodo.5862277. For information on fungal guilds, the FunGUILD database was used (Nguyen, N. H. et al. FUNGuild: An open annotation tool for parsing fungal community datasets by ecological guild. Fungal Ecol. 20, 241–248 (2016)).
Sampling strategy	the 86 grassland plots were chosen to best represent Swiss grassland management and to have a sample size that was logistically feasable. See below, "Data collection" for details on sampling strategy.
Data collection	In 2020 and 2021, intensive field and lab work were carried out to measure the 22 ES-indicators (Figure 1), presenting twelve ecosystem services (ES) according to the CICES typology 29. Regarding the measurement of ES-indicators, only the most relevant information is given here. Further details on these measurements and related analyses can be found in Supplement 2, S2.3. The respective units of the measured ES-indicators are given in Supplement 1, Table S1.3.<br>In June 2020, a first soil sampling campaign was conducted to measure heavy metals, organic carbon stocks, microbial biomass carbon, and to determine the proportions of fungal guilds. Per plot, 20 soil samples along two 20 m transects were taken to a depth of 20 cm and pooled for subsequent analysis. Copper and zinc concentrations were analyzed from 2-mm sieved and air-dried soil using ICP-OES (5110 VDV ICP-OES, Agilent, Santa Clara, CA, US), divided by the respective reference values for Swiss soils, and the highest value of the two metal concentrations per plot was used for the ES-indicator heavy metals. Soil organic carbon was measured

via sulfo-chromic oxidation 70, and carbon stocks were calculated by multiplying organic carbon concentration with bulk density from 5-10 cm, which was measured as described below. Microbial biomass carbon was determined via chloroform fumigation 71,72. For determination of the proportion of fungal guilds, specifically proportion of arbuscular mycorrhizal fungi (AMF) DNA, plant pathogenic fungi, and iconic fungi, DNA extracted from the soil samples was used for sequencing the fungal ITS region on an Illumina platform (Illumina, San Diego, CA, United States). DNA extraction, sequencing and bioinformatic processing was performed following Longepierre et al. (2021) 73, but see Supplement 2, S2.3 for details. Information about fungal guild membership of fungal taxa was identified using FunGUILD 74 for AM fungi and plant pathogenic fungi. Taxa belonging to CHEGD taxa, which include the often particularly colorful grassland macrofungi of high conservational value 75, were identified as indicator for iconic fungi.

In August and September 2020, a second soil sampling campaign was carried out to measure root biomass, bulk density for soil compaction, and soil surface phosphorus concentrations. Root biomass was assessed by washing and sieving soil cores from 0-5 cm depth, from three pooled samples per parcel. To determine bulk density, the fine soil stock (FSS, g cm3) was calculated according to Poeplau et al. (2017) 76 and used together with clay content to calculate packing density 77 as a measure for soil compaction, which is closely related to infiltration capacity, using three pooled soil samples from 5-10 cm depth per plot. For surface soil phosphorus (P) concentrations, we used 10 pooled shallow soil samples (1.5 cm deep) per plot, representing the stratum of soil P which is particularly at risk of erosion and thus depicts a potential eutrophication risk for freshwater ecosystems. Water-extractable soil phosphorus was measured photometrically (Evolution 220 with Cetac ASX-520, Thermo Fisher Scientific, Waltham, MA, United States).

Between the beginning of May and mid-June 2021, vegetation and earthworm surveys were conducted. For vascular plant species richness, all plant species occurring at two 2 m × 2 m quadrats (20 m apart from each other, each 10 m from the plot center) were recorded and summed for a total richness in plant species. The number of edible plant species was calculated based on the vegetation survey (the two 2 m × 2 m quadrats) and literature information 78–80. Potential nectar provision was estimated using the cover of plant species from the vegetation surveys and data on nectar provision per species from the literature 81,82. The number of agricultural weed plants (or of dense patches for clonal plants) was recorded along two transects per site. Leaf damage by herbivorous arthropods was assessed by sampling leaves in the field; one legume, grass, and herb leaf each (if available), every 50 cm along two perpendicular 20 m transects. subsequent visual examination of damage, and percentage of damaged leaves was used as the ES-indicator. Aboveground plant biomass was sampled repeatedly on the plots. In pastures, grazing exclusion cages were installed prior to grazing, and biomass could be sampled at 2 cm above the surface. The biomass sample taken closest to the first date of use as indicated by the farmers in a management survey was analyzed for digestibility (i.e., digestible organic matter) via enzymatic digestion in rumen fluid according to Tilley and Terry (1963) 83. For aboveground biomass yield, vegetation biomass was sampled close to a reference date set to end of May (day of year, DOY, 146). As some plots were sampled later or earlier, either due to displacement of the grazing exclusion cages by cow activity or to logistic reasons, biomass was corrected for sampling date. To this end, biomass weight was divided by the temperature-degree sum until sampling date following the approach described in Menzi et al. (1991) 84. Symbiotic N2 fixation in biomass harvested close to DOY 146 was calculated based on the aboveground biomass as described above and by considering identity and cover of occurring legume species. N content of the legume species occurring on each plot was measured and used together with the modeled mass percent of the respective legume and the biomass yield measure to calculate an index for N fixation (Supplement 2, S2.3). To assess earthworm abundances, three soil pits (30 x 30 x 30 cm) were dug out and the excavated material was checked manually, and number of earthworm individuals were counted.

Aesthetic appreciation of the plant community was derived from standardized pictures of each plot taken prior to the vegetation surveys. An online survey asking people for their personal perception of the aesthetic quality of the respective grassland plant community on a 5-point Likert scale from attractive to unattractive was set up with QuestionPro (QuestionPro Inc, Austin, TX, United States), and widely distributed over e-mail and social media, with finally 521 respondents. The mean aesthetic rating per plot was used as a value of aesthetic appreciation. N2O emissions were calculated according to the IPCC guidelines 85, using fertilizer data from the management interviews and Switzerland-specific information on livestock 69 to estimate the amount of N excreted by grazing animals. Information on N inputs was also used to estimate potential nitrate leaching using a tool developed by the UK

A management survey was conducted in January/February 2021 and 2022 in order to collect detailed information on the management of each investigated grassland plot. The information included grazing dates, number, age, and type of animals, as well as timing, amounts and nature of fertilizer applications. The grazing information was used to calculate the average livestock unit days ha-1 year-1 for each plot over the two years. From the information about amount and type of fertilizer, the total plant-available fertilizer N ha-1 year-1 was calculated based on information from Richner et al. (2017) about available N contents of the different organic fertilizers. Mineral fertiliser N was set to 100% available.

Environment Agency accounting for fertilizer N and animal excreta as sources for nitrate leaching.

Franziska Richter and student helpers collected and recorded the data.

Timing and spatial scale	Spatial scale: Across the Canton of Solothurn, Switzerland. Timing: Soil sampling was carried out between June and September 2020, the other variables were measured between March and June 2021. Measurements were not repeated. The rationale for the timing was to sample within the vegetation period and within a short time frame to allow for comparability between the sites. For the vegetation biomass sampling, (march to June) the rationale was to capture plant growth up to peak production. Originally, all sampling was planned to be in march to May 2020, but due to the pandemic sampling had to be split over two years, with the soil sampling in 2020 and other measurements in 2021.
Data exclusions	No Data was excluded
Reproducibility	Steps taken to ensure reproducibility: use of simple and easy to implement methods for most of the ecosystem services, exact documentation in material and methods, detailed description of plot selection process, use of standard Swiss guidelines for calculation of nitrogen input, availability of code
Randomization	Grasslands stratified random according to the sampling design factors. Samples on the grasslands were taken at specified points along transects that were established in the beginning of the study randomly.
Blinding	Blinding was not relevant for this study

Did the study involve field work? ☒ Yes ☐ No

Field work, collection and transport

Field conditions	Sampling was conducted during the growing season, in sunny as well as in rainy weather but never in temperatures below freezing, as this would have made soil sampling impossible. For all measures except earthworm presence, no influence of rain vs. sun was to be expected (vegetation species survey, biomass sampling, soil sampling), so soil moisture was also measured and earthworm abundances later corrected statistically for soil moisture.
Location	The Canton of Solothurn, Switzerland. Detailed site locations cannot be disclosed, as this would infringe on the privacy of the respective farmers and land owners.
Access & import/export	Access was granted by land owners; no import/export for this study (NA)
Disturbance	no major disturbance was done; minimal disturbance was caused by soil sampling, but care was taken to replace sods (e.g. in earthworm sampling)

Reporting for specific materials, systems and methods

We require information from authors about some types of materials, experimental systems and methods used in many studies. Here, indicate whether each material, system or method listed is relevant to your study. If you are not sure if a list item applies to your research, read the appropriate section before selecting a response.

Materials & experimental systems

n/a	Involved in the study
☒	☐ Antibodies
☒	☐ Eukaryotic cell lines
☒	☐ Palaeontology and archaeology
☒	☐ Animals and other organisms
☒	☐ Clinical data
☒	☐ Dual use research of concern
☐	☒ Plants

Methods

n/a	Involved in the study
☒	☐ ChIP-seq
☒	☐ Flow cytometry
☒	☐ MRI-based neuroimaging

Dual use research of concern

Policy information about dual use research of concern

Hazards

Could the accidental, deliberate or reckless misuse of agents or technologies generated in the work, or the application of information presented in the manuscript, pose a threat to:

No	Yes	
☒	☐	Public health
☒	☐	National security
☒	☐	Crops and/or livestock
☒	☐	Ecosystems
☒	☐	Any other significant area

Experiments of concern

Does the work involve any of these experiments of concern:

No	Yes	
☒	☐	Demonstrate how to render a vaccine ineffective
☒	☐	Confer resistance to therapeutically useful antibiotics or antiviral agents
☒	☐	Enhance the virulence of a pathogen or render a nonpathogen virulent
☒	☐	Increase transmissibility of a pathogen
☒	☐	Alter the host range of a pathogen
☒	☐	Enable evasion of diagnostic/detection modalities
☒	☐	Enable the weaponization of a biological agent or toxin
☒	☐	Any other potentially harmful combination of experiments and agents
